# Small Fragment Homologous Replacement: Evaluation of Factors Influencing Modification Efficiency in an Eukaryotic Assay System

**DOI:** 10.1371/journal.pone.0030851

**Published:** 2012-02-16

**Authors:** Andrea Luchetti, Antonio Filareto, Massimo Sanchez, Giampiero Ferraguti, Marco Lucarelli, Giuseppe Novelli, Federica Sangiuolo, Arianna Malgieri

**Affiliations:** 1 Department of Biopathology and Diagnostic Imaging, Medical Genetics Section, School of Medicine, Tor Vergata University, Rome, Italy; 2 Department of Cell Biology and Neurosciences, Istituto Superiore di Sanità, Rome, Italy; 3 Department of Cellular Biotechnologies and Hematology, Sapienza University of Rome, Rome, Italy; 4 National Agency of Evaluation for University and Research (ANVUR) and Ospedale San Pietro Fatebenefratelli, Rome, Italy; University of Minnesota, United States of America

## Abstract

Homologous Replacement is used to modify specific gene sequences of chromosomal DNA in a process referred to as “*Small Fragment Homologous Replacement*”, where DNA fragments replace genomic target resulting in specific sequence changes. To optimize the efficiency of this process, we developed a reporter based assay system where the replacement frequency is quantified by cytofluorimetric analysis following restoration of a stably integrated mutated eGFP gene in the genome of SV-40 immortalized mouse embryonic fibroblasts (MEF-SV-40). To obtain the highest correction frequency with this system, several parameters were considered: fragment synthesis and concentration, cell cycle phase and methylation status of both fragment and recipient genome. In addition, different drugs were employed to test their ability to improve technique efficiency. SFHR-mediated genomic modification resulted to be stably transmitted for several cell generations and confirmed at transcript and genomic levels. Modification efficiency was estimated in a range of 0.01–0.5%, further increasing when PARP-1 repair pathway was inhibited. In this study, for the first time SFHR efficiency issue was systematically approached and in part addressed, therefore opening new potential therapeutic *ex-vivo* applications.

## Introduction

*In situ* modification by gene targeting approach allows the recovery of a normal gene function [1], offering significant advantages compared to gene augmentation. Mutated genetic instructions are site-specifically modified in long-term and genetically inheritable manner, maintaining their native sequence context. By this way, targeted gene results modulated by the endogenous regulatory machinery, thus maintaining physiologic expression pattern. In mitotic cells, homologous recombination (HR) is a basic mechanism to repair DNA damage and in particular DNA double-strand breaks (DSBs). Two main issues hamper easy gene targeting in vertebrate cells: the low frequency of HR events, generally occurring once every 10^5^–10^7^ treated cells, and the high rate of random (non-homologous) integrations, that occur approximately once every 10^2^–10^4^ treated cells. Among different gene targeting strategies currently employed in laboratory, Small Fragment Homologous Replacement (SFHR) uses Small DNA Fragments (SDFs) to obtain homologous replacement in recipient cells [2]. Once within cells, SDFs trigger the exchange between their sequences and the genomic DNA [3] through a still undefined mechanism [4]. It is likely that the fragment recognizes and anneals to its homologous target, promoting the formation of a D-loop structure. This hybrid structure could activate the endogenous machinery involved in DNA repair and, by HR, allow the SDF to be integrated into the genomic DNA [5]. SFHR was successfully used to target genomic mutations with different features, working *in vitro* and *in vivo* in both human and mouse cells, demonstrating its ability to correct several disease-associated genes [6], such as: *Cftr* [7]–[11] (Cystic Fibrosis), *Dystrophin* [12], [13] (Muscular Dystrophies), *SMN* [14], [15] (Spinal Muscular Atrophy), *DNA-PKs* [16] (SCID), *HPRT* [17] and *β-globin* [18] (β–thalassemia). Importantly, the SFHR-mediated DNA modification has been shown to properly target genomic DNA in both differentiated and undifferentiated stem cells [18], resulting in long-term correction through clonal expansion.

Among factors influencing targeting mechanism, changes in the chromatin structure during cell cycle, as well as cell mechanisms involved in genome structure maintenance, are key factors in SFHR efficiency [19]–[21]. Moreover, epigenetic changes were detected after *in vitro* gene targeting of stem cells [22]. Together these evidences strongly suggest functional interconnections between molecular mechanisms controlling chromatin structure, cell cycle, DNA methylation, DNA repair and gene targeting.

To date, studies linking SFHR to epigenetic modifications or to cell cycle are still missing. Even if the potential of SFHR is promising, it is currently limited by low and variable frequency of correction, ranging from 0.01% to 5% *in vitro* and about 0.1% *in vivo* [2]. Furthermore the absence of a selectable marker makes difficult to quantify and optimize the efficiency of SFHR-mediated modifications. In this study we developed an *in vitro* reporter assay system able to properly quantify the percentage of SFHR-modified cells. A mutated non-fluorescent eGFP gene was stably integrated within genomic DNA of immortalized murine embryonic fibroblasts. Transfected SDFs were homologous to eGFP wild-type sequence, allowing reporter fluorescence recovery. The aim of this work was to evaluate the influence that specific cellular mechanisms could have on SFHR efficiency, in order to increase technique efficacy. Several experimental variables were investigated such as SDF structure, cell cycle and DNA methylation of both SDF and recombinant host genome.

Increased replacement efficiency will be useful for further *ex vivo* SFHR gene therapy applications.

## Results

### Clones construction and eGFP genomic integration

**Figure 1:**
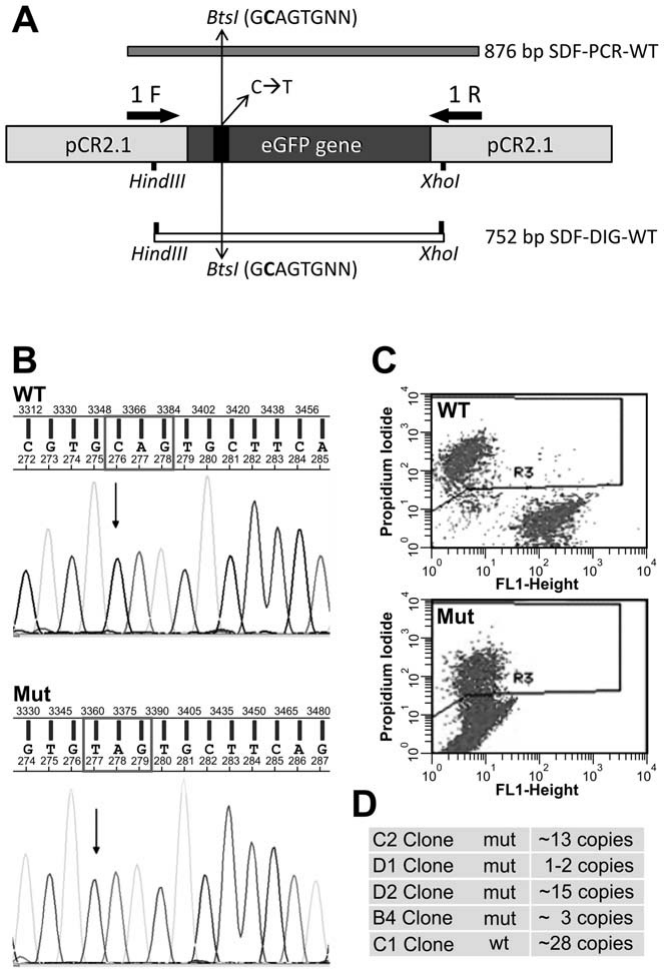
Experimental design for SDF and cell clone generation. A) SDF sequence is homologous to the entire wild type eGFP coding sequence. SDF-PCR-WT, 876 bp long was generated by PCR amplification with primer pair 1F/1R (Table 1). SDF-DIG-WT, 752 bp long, was obtained by *HindIII* and *XhoI* digestion of pCR-2.1 vector. C/T transition, responsible of fluorescence switching off, is showed. B) Sequencing analysis showing wild type (WT; top panel) and mutated (Mut; bottom panel) pCEP4-eGFP in C1 and D1 cell clones, respectively. Arrows indicate the modified base (C→T). C) FACS density plot of C1 (WT; top) and D1 (Mut; bottom) respectively. D) pCEP4-eGFP copy number determination for each cell clone.

*In vitro* mutagenesis was carried out on pCEP4 residue 210 located in the coding region of wt eGFP gene. The glutamine (CAG) to stop codon (TAG) transition causes, at the same time, a fluorescence switch off and a *BtsI* restriction site disruption (Fig. 1A). Successively SV-40 immortalized MEF were transfected with linearized either wild type (pCEP4/wt-eGFP) or mutated (pCEP4/mut-eGFP) plasmids. Clonal dilution and hygromycin selection were performed to obtain homogeneous transgenic cell lines, stably integrating wild type or mutated copies of eGFP gene, as demonstrated by sequencing (Fig. 1B) and FACS analyses (Fig. 1C). For each clone pCEP4/eGFP copy number was determined by Taqman qPCR (Fig. 1D). Genomic DNA and cDNA amplification followed by *BtsI* enzymatic digestion confirmed the presence of the inserted mutation in all mutated clones (data not shown). Moreover FISH analysis on D1 clone demonstrated the genomic integration of the pCEP4/mut-eGFP vector (Fig. S1). Among four mutated cell clones, D1 was employed for all the experiments because containing only one copy of the transgene. D1 represented our assay system in which different parameters were tested, in order to quantify the efficiency of gene modification.

### Transfection parameters setting

**Figure 2:**
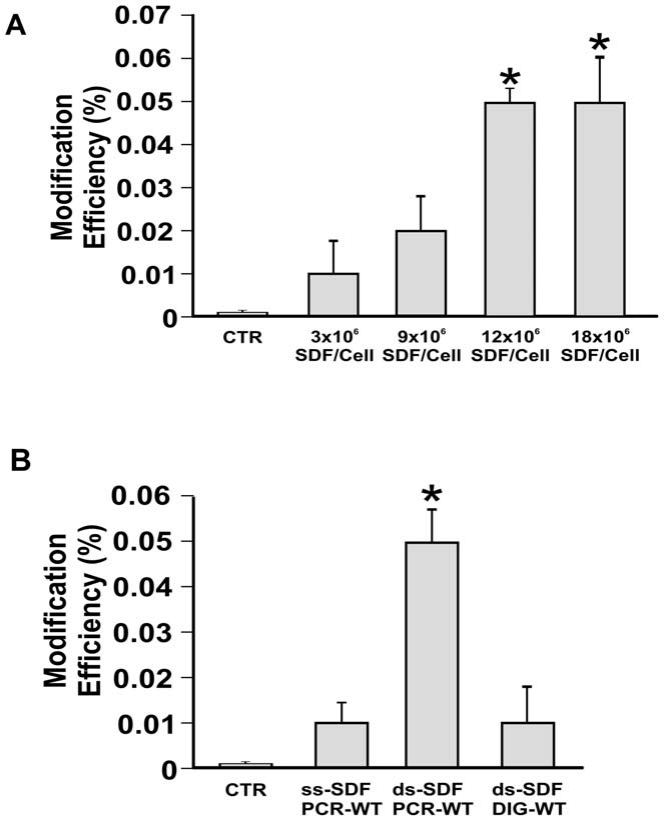
Amount and type of transfected SDF. A) Correction efficiencies after transfecting different amounts of SDF-PCR-WT in D1 cells. Positive events are used to determine the overall modification efficiency respect to D1 control cells transfected with a SDF homologous to mutated eGFP sequence (CTR). B) Different kind of SDFs were tested in D1 cells: double (ds-SDF-PCR-WT 12×10^6^ SDF/cell) or single strand (ss-SDF-PCR-WT 12×10^6^ SDF/cell) PCR fragments and fragment obtained by enzymatic digestion (ds-SDF-DIG-WT 12×10^6^ SDF/cell) were compared to cells transfected with SDF homologous to mutated eGFP sequence (CTR). For representative FACS dot plots see Fig. S3 and S4.

After optimization of transfection conditions (Fig. S2 and Information S2), SDF concentration was tested: 1.7×10^6^ unsynchronized cells were transfected with increasing amounts of SDF-PCR-WT ranging from 5 µg (3×10^6^ SDF/cell) to 30 µg (18×10^6^ SDF/cell) (Fig. 2A). Targeted correction rates were measured by flow cytometry 3 days after transfection. The best efficiency (0.05%, *p = 0.00002) was obtained using 12×10^6^ molecules of SDF/cell (20 µg) (Fig. 2A and Fig. S3). This amount has been used for all further transfections. Higher SDF concentrations (18×10^6^ SDF/cell) were also tested eliciting increased cell mortality (data not shown).

We then evaluated three different experimental protocols for SDFs synthesis, relating them to correction efficiency. Specifically a SDF-PCR-WT fragment, 876 bp long, either double (ds) or single stranded (ss), obtained by enzymatic amplification, and a SDF-DIG-WT fragment, 752 bp long, obtained by digestion of pCR-2.1 vector was used (Fig. 1A). Three days after transfection, a correction frequency of 0.05% (*p = 0.001) was detected by FACS when ds-SDF-PCR-WT was used, resulting five-folds higher than ds-SDF-DIG-WT (0.01%, p<0.07) (Fig. 2B and Fig. S4). The repair frequency obtained using a control SDF (CTR), homologous to mutated eGFP sequence, was essentially identical to background (about 0.00001%). Heat denatured ss-SDF-PCR-WT fragment produced an efficiency of 0.01%, equivalent to that obtained by SDF-DIG-WT (Fig. 2B, p = 0.07).

If not differently stated, the SDF hereafter used in this work is always double stranded.

### Cell cycle, SDF methylation and modification efficiency

Manipulation of cell cycle progression by inducing DNA damage has recently been shown to be one factor governing the frequency of the targeted gene repair reaction [16].

**Figure 3:**
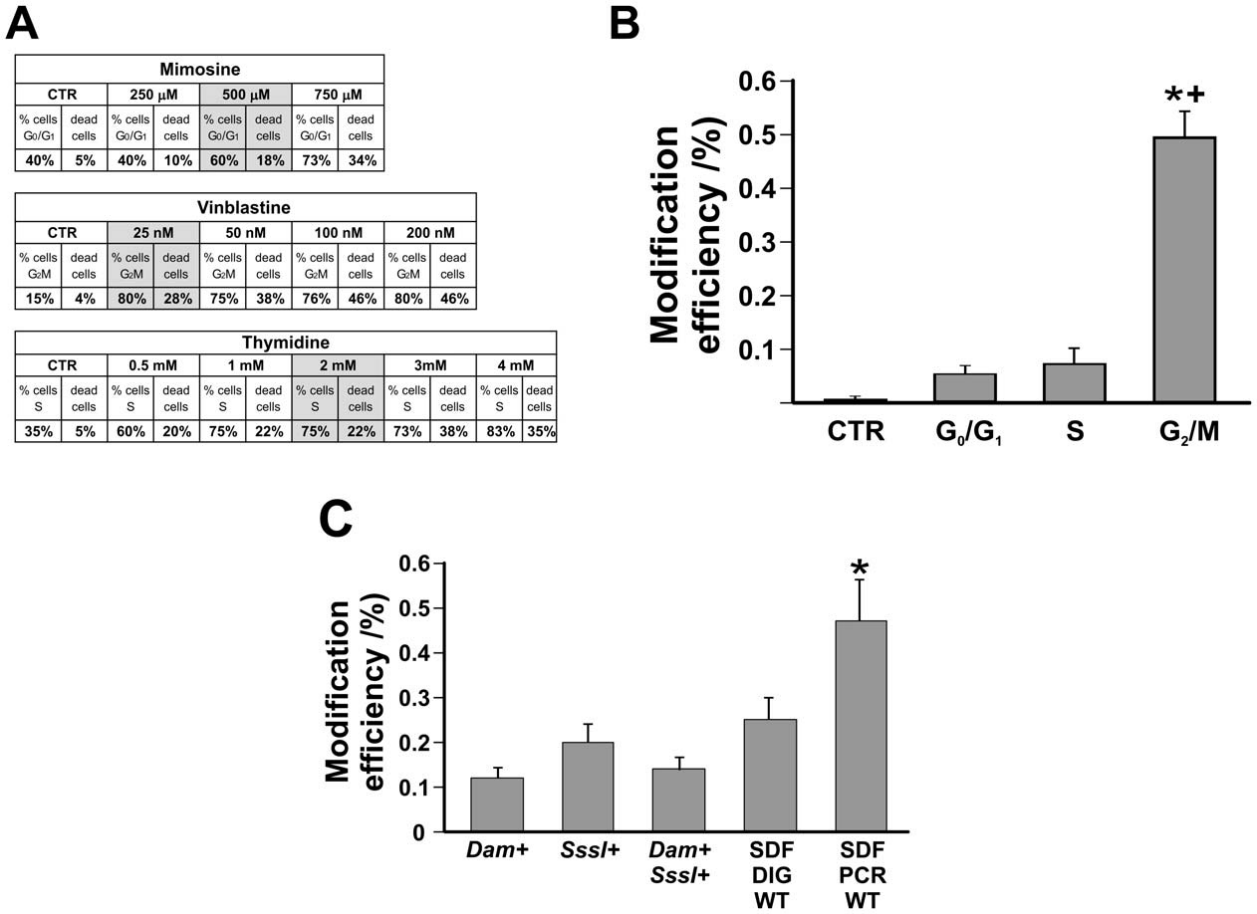
Modification efficiencies obtained testing different concentrations of mimosine, thymidine, vinblastine and SDFs with different superimposed methylation patterns on D1 cells. A) For each drug the concentration that gives the highest percentage of synchronized cells and the lowest cell death (highlighted in grey) was selected. B) Correction efficiencies after transfection in different cell cycle phases. A SDF homologous to mutated eGFP sequence was used as control (CTR). Gene modification efficiency was enhanced when cells are synchronized in G_2_/M phase (*p = 0.0001 respect to CTR and +p = 0.0001 respect to unsynchronized cells, Fig. S5). C) Differently *in vitro* methylated SDFs were tested to assess methylation involvement in gene modification efficiency. SDF-PCR-WT gave the highest efficiency of modification (*p resulted to be significant when compared to all treatments; specifically p = 0.002 respect to *Dam+*, p = 0.01 respect to *SssI*+, p = 0.008 respect to *Dam+/SssI+*, and p = 0.009 respect to SDF-DIG-WT). For representative FACS dot plots see Fig. S5 and S6.

To determine whether cell cycle phase might affect the efficiency of gene repair, we evaluated gene targeting in cell populations enriched in G_0_/G_1_, S, and G_2_/M phases. Cell synchronization was previously optimized in order to obtain an high cell cycle enrichment together with an high cell viability (Fig. 3A, grey columns). Best synchronization conditions were evaluated by flow cytometry using propidium iodide (PI), and soon after used to treat cells before transfecting 12×10^6^ molecules/cell of SDF-PCR-WT (Fig. 3B and Fig. S5). Fluorescence was quantified 72 hours after transfection to exactly determine phase-specific gene repair frequencies. Compared to unsynchronized cells (Fig. 2B and Fig. S4; 0.05%), G_2_/M synchronized cells showed an increased correction efficiency to 0.5% (*p = 0.0001 respect to CTR and **+**p = 0.0001 respect to unsynchronized cells). No significant differences in modification efficiencies were detected comparing synchronized with unsynchronized cells in G_0_/G_1_ (0.05%, p = 0.001 respect to CTR and n.s. respect to unsynchronized cells) or in S phase (0.07%, p = 0.0008 respect to control and n.s. respect to unsynchronized cells) (Fig. 3B and Fig. S5). CTR column indicates synchronized cells transfected by SDF homologous to mutated eGFP sequence.

To test the hypothesis about the influence of SDF methylation on SFHR efficiency, differently methylated SDFs were produced *in vitro* using *SssI* or *Dam* or both DNA-methyltransferases, and then transfected into G_2_/M synchronized cells (Fig. 3C, Fig. S6 and Fig. S7 and Information S2). The efficiency of SDF replacement was up to 80% lower (*Dam^+^* methylation; 0.12%) than that obtained transfecting SDF-PCR-WT (0.48%), where no methylation was present. A reduction of about 50% was observed when SDF-DIG-WT, harboring prokaryotic methylation (Fig. S7), was used (0.22% vs 0.48%; Fig. 3C and Fig. S6).

### Cell sorting and molecular analysis

**Figure 4:**
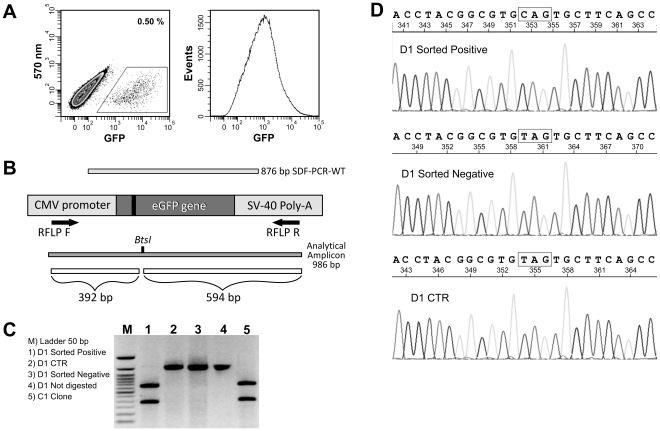
Molecular analyses of sorted D1 cells. A) Modification efficiency in D1 cells transfected with 12×10^6^ SDF-PCR-WT/cell. Positive cells (0.5%) were sorted and soon after reanalyzed (right panel) to asses population purity (>99%). B) PCR/RFLP analysis design. C) Amplicon is generated using RFLP primer pair. Cells transfected with mutated SDF represent our control (D1 CTR, lane 2). All amplification products were digested with *BtsI*, except lane 4. Restriction patterns of Sorted positive D1 clone (lane 1) and of parental eGFP C1 cells (lane 5) were identical. No restriction bands were present in D1 CTR (lane 2) and in sorted negative cells (lane 3). M is ladder 50 bp. D) Sequence analysis of D1 cells (sorted positive, sorted negative and CTR). The site-specific T-to-C conversion was present only in sorted positive cells.

SDF-PCR-WT (12×10^6^ molecules/cell) was transfected into G_2_/M synchronized cells and fluorescent events were sorted (Fig. 4A), FACS reanalyzed (to check cell population purity) and placed in culture for several passages (about 10). Molecular analyses were performed on a sorted cell population to confirm genomic modification and its persistence over time. RFLP analysis was performed on a 986 bp amplicon using *BtsI* enzyme, whose restriction site has been recovered as result of successful SDF replacement. Primers design (RFLP F and RFLP R; Fig. 4B and Table 1) allowed the amplification of both wild-type and mutant eGFP sequence, avoiding randomly integrated or free SDFs. Sorted positive D1 clone restriction pattern (Fig. 4C, lane 1) was clearly comparable to parental C1 clone in which wt eGFP sequence was present (Fig. 4C, lane 5). No restriction bands were present in CTR (Fig. 4C, lane 2; in which a SDF homologous to mutated eGFP sequence was transfected) and in sorted negative non fluorescent cells (Fig. 4C, lane 3), indicating no correction. Direct sequencing of the analytical amplicon demonstrated the presence of wild type base (cytosine C) at position 210 of the coding region in D1 sorted positive cells (Fig. 4D). Thymine (T), belonging to the mutated gene, was present in D1 sorted negative and D1 CTR DNA. No other base alteration was evidenced. Allelic discrimination by Real Time PCR was also performed confirming previous results (Fig. S8).

**Table 1:** Sequence and characteristics of primers.

Name	Sequence	Annealing	Amplicon	Fragment
Mutagenesis f	CCTACGGCGTGTAGTGCTTCAGC	55°C		
Mutagenesis r	GCTGAAGCACTACACGCCGTAGG			
eGFP f	CTGCTGCCCGACAACCA	60°C	74 bp	
eGFP r	ATGTGATCGCGCTTCTCGTT			
eGFP probe	6FAM-CCAGTCCGCCCTGAGCAAAG-TAMRA			
ApoB f	CACGTGGGCTCCAGCATT	60°C	74 bp	
ApoB r	TCACCAGTCATTTCTGCCTTTG			
ApoB probe	VIC-CCAATGGTCGGGCACTGCTCAA-TAMRA			
RFLP f	CACTGCATTCTAGTTGTGGTTTGT	55°C	986 bp	
RFLP r	CACCAAAATCAACGGGACTT			
1f	ACTCATCAATGTATCTTATCAT	55°C	876 bp	SDF-PCR-WT
1r	AGGTCTATATAAGCAGAGCT			
5f	CGTAAGTTATGTAACGCGGAACTC	64°C	167 bp	St3
5r	GGCCATTGCATACGTTGTATC			
6f	GTAGGTCAGGGTGGTCACG	64°C	699 bp	c
6r	GTAACGCCAATAGGGACTTTCC			
7f	AACTTGTTTATTGCAGCTTATAATGG	64°C	383 bp	d
7r	AGATCCGCCACAACATCG			
8f	AAAACCTCTACAAATGTGGTATGG	64°C	503 bp	e
8r	CATAAAGGCAATGTTGTGTTGC			
8f1	GGGGACCAAACACAAAGG	64°C	138 bp	St4
8r	CATAAAGGCAATGTTGTGTTGC			

**Figure 5:**
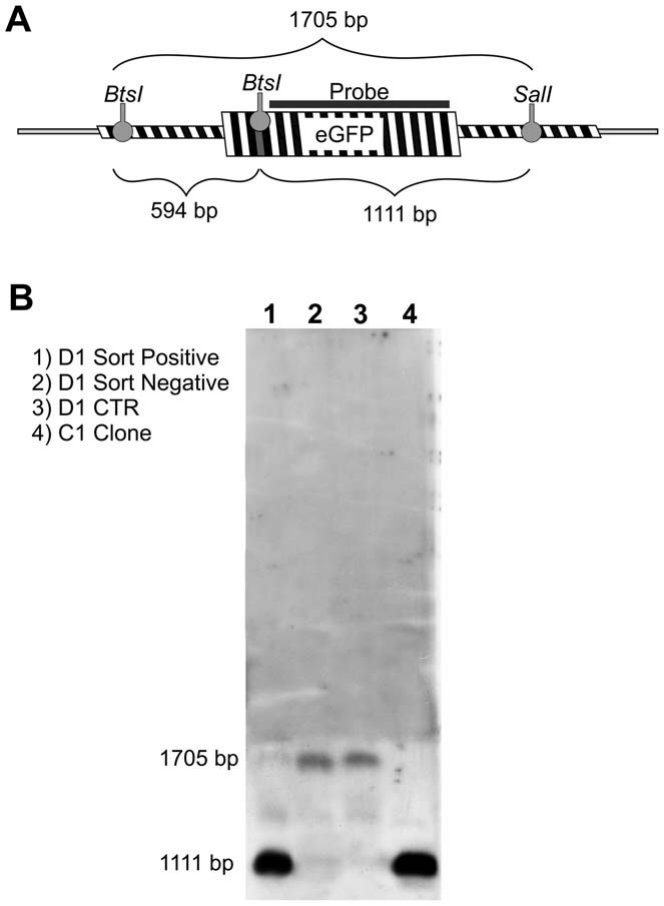
Southern blot analysis. A) Probe design. A 566 bp probe was used, recognizing a region of eGFP gene. Dashed box correspond to pCEP4-eGFP locus integrated within genomic DNA. *BtsI* site recovery highlight the correction of the eGFP gene. After *SalI/BtsI* genomic DNA digestion, two different restriction pattern can be obtained, according to the presence/absence of *BtsI* restriction site. B) Southern blot. A 1111 bp band was obtained only in cells in which *BtsI* site is present (D1 sorted positive and C1 clone).

Southern blot analysis was performed to further assess genomic modification (Fig. 5). D1 sorted positive and negative cells were compared to control cells (D1 CTR) and to parental C1 fluorescent clone. *SalI/BtsI* digested genomic DNA was probed to a 566 bp DNA fragment homologous to the SDF (Fig. 5A). As expected, in SFHR-modified cells (Fig. 5B, lane 1) and in the C1 clone (Fig. 5B, lane 4) only the 1111 bp band was clearly detectable, demonstrating *BtsI* site recovery in D1 sorted positive cells. A higher band of 1705 bp was present in D1 non-fluorescent (Fig. 5B, lane 2; negatively sorted) and in D1 CTR (Fig. 5B, lane 3; transfected with SDF homologous to mutated eGFP sequence).

### Genomic DNA methylation involvement in the inactivation of eGFP expression

**Figure 6:**
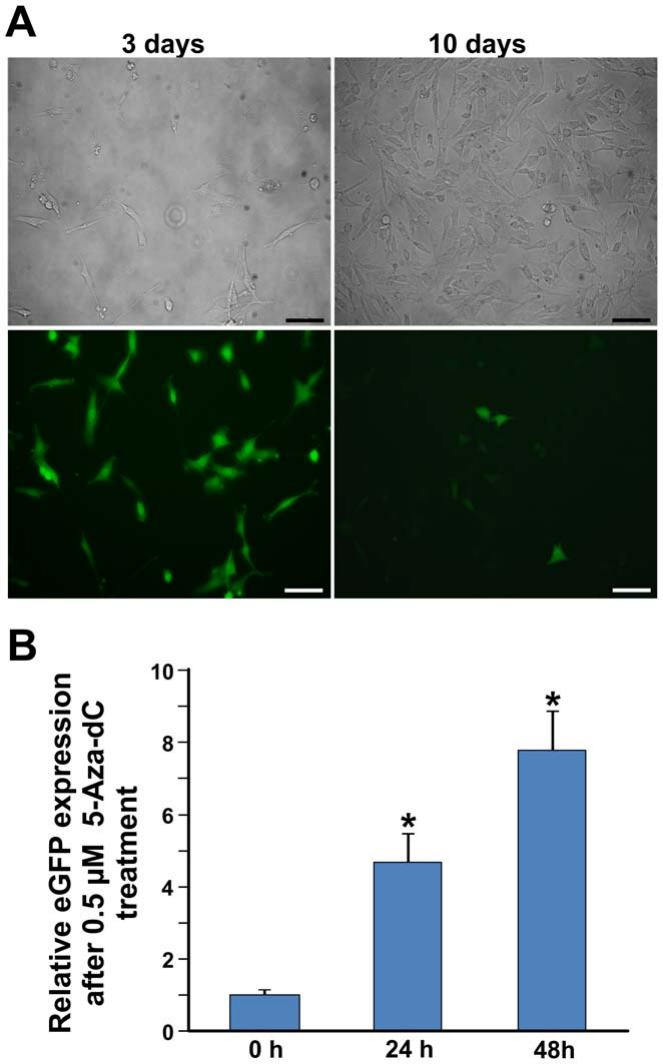
eGFP expression increased after 5-Aza-2′-Deoxycytidine treatment. A) Bright field (upper row) and fluorescent (bottom row) images of D1 sorted corrected cells at different experimental time (scale bar: 150 µm). B) eGFP expression, analyzed by Real Time PCR, after 24 h and 48 h of treatment with 0.5 µM 5-Aza-2′-Deoxycytidine respect to untreated (0 h) (*p = 0.002); untreated cells, at 24 h and 48 h, usually showed a decreasing relative eGFP expression (data not shown).

**Figure 7:**
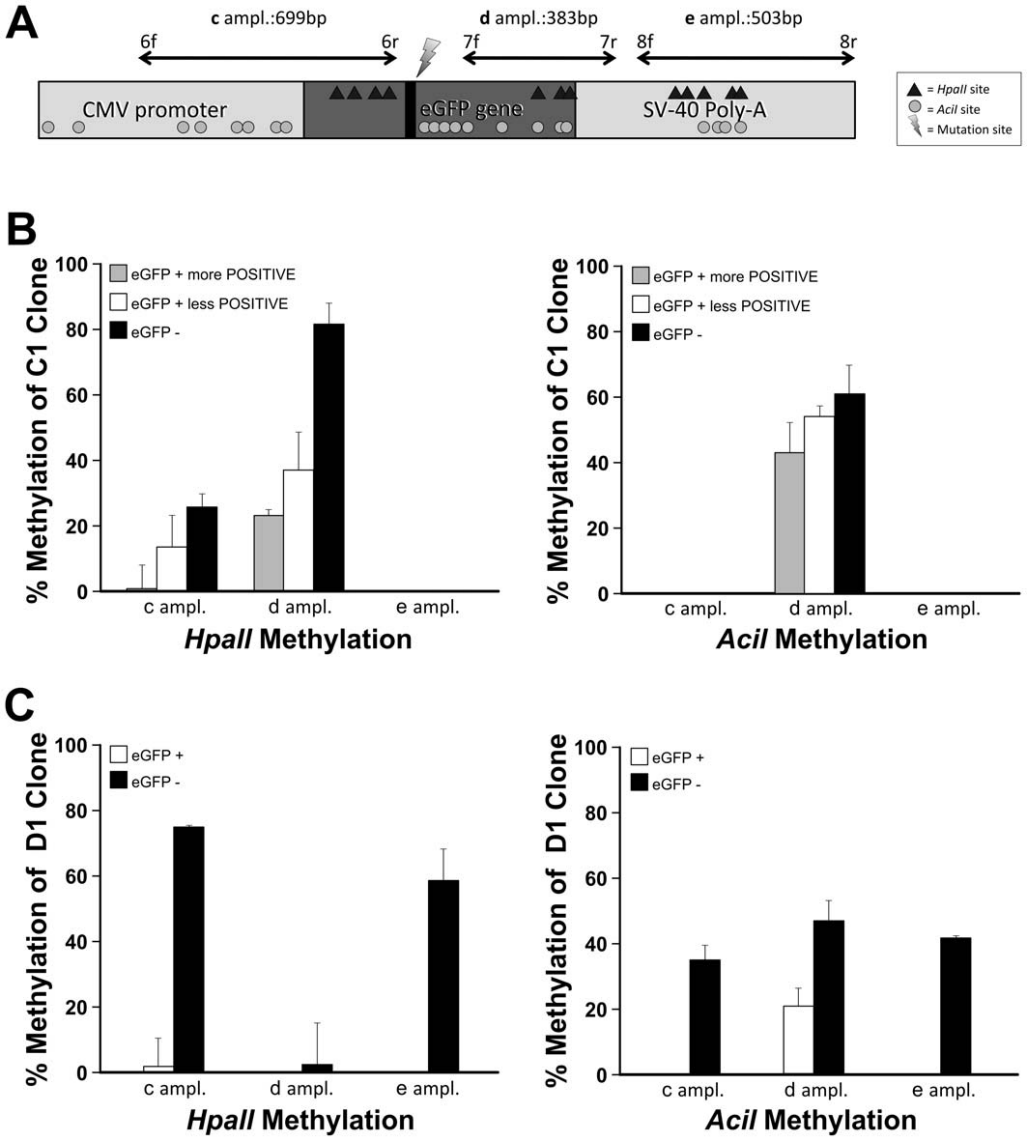
*HpaII* and *AciI* methylation analyses of integrated eGFP in C1 and D1 clones. A) Experimental design showing the amplicon regions and their length within eGFP locus integrated in genomic DNA. *HpaII* and *AciI* site are indicated. B) Densitometric analyses of parental C1 clone methylation pattern on eGFP^+^ more positive, eGFP^+^ less positive and eGFP^−^ cells (see also Fig. S9B and Fig. S10B). ANOVA test gave a statistical significance of p<0.001 and p<0.005 respectively for *HpaII* and *AciI* panels. C) Densitometric analysis of methylation pattern of D1 SFHR-modified clone on both fluorescent and non fluorescent cells (see also Fig. S9C and S10C). ANOVA test gave a statistical significance of p<0.001 for both panels.

By culturing D1 fluorescent cells, a gradual loss of eGFP expression was noticed (Fig. 6A). A similar trend was observed in parental C1 (data not shown). Retro-mutation was excluded by both RFLP and sequencing analyses, confirming the presence of SDF-mediated C nucleotide, regardless to cell fluorescent phenotype (data not shown). To assess DNA methylation involvement in the eGFP expression, D1 SDF-modified cells were resorted. eGFP sorted negative cells (but still carrying the correction) were treated for 24 and 48 hours with 0.5 µM of 5-Aza-2′-Deoxycytidine (5-Aza-dC). eGFP expression, monitored by Real Time-PCR (Fig. 6B), showed more than a four-fold increase after 24 hours (*p = 0.002). The expression further doubled after 48 hours of treatment, when compared to untreated cells (*p = 0.002). Untreated cells usually showed a decreasing relative eGFP expression according to fluorescence decrement (data not shown). These results demonstrated that the percentage of SDF-mediated modification in transfected cells was underestimated, because of a methylation-mediated silencing of eGFP expression. To investigate the correlation between the eGFP locus methylation status and its time-dependent expression, studies by multiplex *HpaII*/PCR and *AciI*/PCR analysis were performed using either C1 parental or D1 SDF-corrected cells (Fig. 7). Three different amplicons (c, d and e), spanning eGFP locus including its promoter, were analyzed (Fig. 7A, S9 and S10 and Information S2). In Figures 7B and 7C the percentage of methylation obtained from densitometric analyses of electrophoretic restriction pattern of the three amplicons is reported (see also Fig. S9, S10). In both C1 fluorescent and non fluorescent sorted populations, the d amplicon (Fig. 7B) resulted the most methylated, either for *HpaII* or *AciI*, while in the e amplicon neither *HpaII* nor *AciI* methylation were evidenced. In the c amplicon the *HpaII* methylation was lower than in d fragment, whereas the *AciI* methylation resulted absent. Importantly always non-fluorescent cells showed the highest levels of both *HpaII* and *AciI* methylation. In the parental C1 clone there is a good correlation between eGFP inactivation and both *HpaII* and *AciI* DNA methylation patterns. The same analysis was carried out on SFHR-modified D1 cells. After sorting, fluorescent cells were placed in culture and, as previously observed, gradually lost fluorescence. A re-sorting of phenotipically heterogeneous corrected D1 cells allowed us to distinguish between eGFP^+^ and eGFP^−^ cells. In fluorescent modified-D1 cells (Fig. 7C) all the analyzed zones showed no or very low levels of both *HpaII* or *AciI* methylation. On the contrary, non fluorescent modified D1 cells showed considerable level of both *HpaII* and *AciI* methylation, with the exception of the d zone that resulted devoid of *HpaII* methylation. Also in D1 modified cells, methylation resulted to be directly correlated with eGFP expression.

### 1,5-Isoquinolinediol drug treatment increase correction efficiency

**Figure 8:**
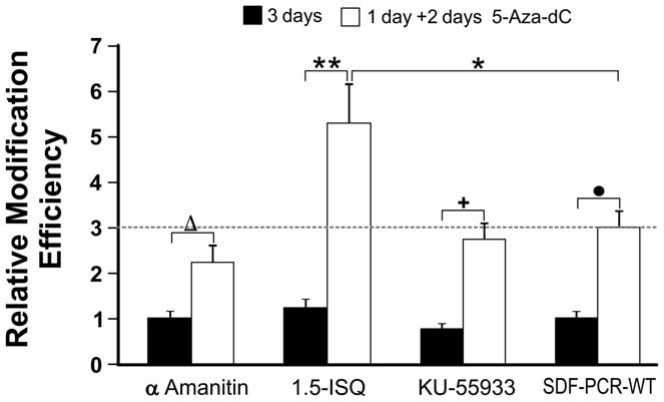
Relative modification efficiency in D1 cells transfected with SDF-PCR-WT and treated with α-Amanitin, 1,5-Isoquinolinediol and KU-55933. Transfected samples were analyzed three days after transfection (3 days; black columns) or in parallel treated 24 hours after transfection with 0.5 µM of 5-Aza-dC for 48 hours (1 day+2 days 5-Aza-dC; white columns). No statistically significant differences were observed at 3 days (black bars) respect to untreated cells (SDF-PCR-WT). Demethylating effect of 5-Aza-dC increased eGFP detection in all samples (white columns) in a statistically significant manner (Δ p = 0.003; +p = 0.01; • p = 0.0007). 5-Aza-dC addition also disclosed the effect of 1,5-Isoquinolinediol on SDF-mediated correction in a statically significant manner respect either to cells not treated with 5-Aza-dC (**p = 0.0002) and to the cells transfected with SDF-PCR-WT in which no drug was added (*p = 0.003). Dashed lines refers to modification efficiency observed in cells without addition of any drug but treated by 5-Aza-dC. Results are from mean values of three independent experiments and are reported as relative modification efficiencies in respect to control without drugs (SDF-PCR-WT).

Finally, three drugs, potentially involved in SFHR mechanism, were tested to verify their effect on correction efficiency. Specifically KU55933, 1,5-Isoquinolinediol (1,5-ISQ) and α-Amanitin were added to transfected cells, that are, respectively, inhibitor of ATM kinase, PARP-1 and RNA polymerase II [23]–[30]. No statistically significant variations in modification efficiency were observed three days after transfection (Fig. 8, black bars) respect to SDF-PCR-WT control sample, in which no drugs were added.

To disclose methyl-hidden correction events, 5-Aza-dC was added to all samples 24 hours after transfection (Fig. 8, white bars), resulting in an overall increase of fluorescence, statistically significant.

When 5-Aza-dC is added to 1,5-ISQ treated cells a statistically significant increase in correction efficiency was obtained both respect to cells untreated with 5-Aza-dC (Fig.8, 1,5 ISQ black bar, **p = 0.0002) and to control PCR (Fig.8, SDF-PCR-WT white bar, *p = 0.003).

These data indicated PARP-1 as a potential SFHR-efficiency modifier.

## Discussion

During last years, gene repair approaches received increasing attention because of their safety compared to traditional gene therapy strategies, where additional copies of therapeutic genes are delivered and expressed in transduced cells [31]–[34].

Gene repair strategies attempt to directly correct endogenous genetic mutations *in situ*, maintaining gene regulation under endogenous promoter control. Targeted gene conversion represents also a tool for functional genomics in order to define gene function. Furthermore, it is particularly intriguing the possibility of gene-targeting in stem cells, that is the primary aim in both therapeutic application and functional genomics [35], [36].

Issues associated with this technique are still numerous and many steps involved in gene correction process are still unknown [3], [37]. Thus it is critical that gene repair becomes an efficient and reproducible strategy before its application to clinical medicine.

In this study, we developed a reporter-based assay system useful to optimize SFHR experimental procedure. A mammalian immortalized cell line was created with a stably integrated eGFP gene. After transfecting SDFs into these cells, FACS analysis allowed to quantitatively assess gene correction efficiency, considering each fluorescent cell as a single correction event.

Several parameters (potentially involved in the SFHR process) were evaluated: SDFs preparation, concentration, methylation and delivery; integrated eGFP gene methylation pattern after correction and, more importantly, the influence of cell cycle in the gene modification process.

Moreover three drugs were tested, whose targets could play a key role in SFHR process.

Optimal SDF concentration was identified. Moreover fragment synthesis by PCR amplification resulted to be preferable to that obtained by plasmid restriction probably because of the methylation pattern. To confirm this aspect, *in vitro* methylated fragments (*Dam*, *SssI* or both) were tested. The low correction efficiencies, obtained using methylated SDFs, could arise from a still unknown mechanism, possibly involving methyl-binding protein that could inhibit their integration within genomic DNA.

Studies on oligonucleotide ssODNs and cell cycle indicated that gene modification frequencies varied considerably between the various cell cycle phases, in particular late S-phase was shown to be the most amenable for gene repair [20], probably due to increased activity of replication forks and to a more favorable chromatin conformation. Transfecting SDFs into cells synchronized in different cell cycle phases, we noticed that replacement efficiencies were highest in vinblastine-enriched G_2_/M-phase cells. Correction takes place also in G_0_/G_1_ or S phase, even if with lower efficiency. SDFs, differently from ssODNs [20], seem to have a greater probability to access the target locus during G_2_/M phase possibly because chromosomes, already replicated (tetraploid status), are still organized in loosely packed fibers.

Southern Blotting analysis suggest that random integration (due to Non-Homologous End Joining of the fragment) is low at best [38], however the possibility that it can occur below the level of detection of the analytical system cannot be dismissed. Regarding genomic methylation, the quantitative levels of methylation of the integrated eGFP locus appeared to be correlated with cell replication, in both parental C1 clone (where wild type e GFP locus is present) and in D1 corrected clone. It should be noted that D1 corrected cells have been sorted twice: soon after transfection (isolating corrected fluorescent from non-corrected non-fluorescent cells), and after *in vitro* fluorescence decrease (isolating in this case two cell populations both corrected: fluorescent and silenced non-fluorescent).

The methylation patterns is however different in C1 and in D1 resorted clones: the first appeared to be more prone to methylate the 3′ end of the SFHR target region (d amplicon), the second preferentially underwent to methylation of regions upstream and downstream the SFHR target region (c and e amplicon). It should be taken into account that this effect may depend on the fact that the correction of D1 clone was achieved by the integration of a non methylated PCR product that can undergo to a slower methylation dynamics during cell replication. However a change in the methylation patterns may specifically arise as consequence of the recombination event possibly recognized by cellular machinery of defense from invading DNA; anyway this leads to gene silencing and consequently to an underestimation of correction events [39]. Even the hypothesis that the differences in methylation patterns between C1 parental cell line and D1 corrected clone are due to the epigenetic background of the genomic zone where the eGFP construct inserted cannot be ruled out. These effects seem to be also site-specific, in fact *HpaII* and *AciI* methylation patterns resulted qualitatively and quantitatively different. These results indicate DNA methylation as an experimental variable to be considered, because partially masking the real efficiency of SDF-mediated correction.

In order to preserve the integrity of the genome, cells have developed various pathways to sense and overcome DNA damage. Environmental factors like radiations or toxins, as well as spontaneous DNA lesions, trigger checkpoint activation and consequent cell cycle arrest allowing DNA repair or leading to apoptosis. Two key proteins are mainly involved in recognition of DNA damage and signal transduction to p53: ATM and PARP-1. PARP-1 participates mainly in base excision repair (BER), whereas ATM mainly in homologous recombination (HR) [40]–[41]. ATM is a protein kinase capable to arrest the cell cycle following DNA damage, thus activating DNA double strand breaks repair machinery [23]–[26] while PARP-1 participates in signaling from DNA single strand lesions. Recently PARP-1 has been linked to the regulation of chromatin structure and transcription, DNA methylation and imprinting, insulator activity, and chromosome organization, playing key roles in a number of nuclear processes [27]–[29]. Moreover, it should be taken into account that transcribed parts of the genome are more efficiently repaired and DNA damage is removed faster from transcribed strands (TS) than non- transcribed strands (NTS) [30]. At this purpose we tested three drugs affecting different proteins that could be potentially involved in the mechanism of SFHR: KU-55933, a specific ATM kinase inhibitor, 1,5-Isoquinolinediol, PARP-1 inhibitor, and α-Amanitin, able to block RNA polymerase II and prevent transcription initiation and elongation. No statistically significant differences were detected treating cells with the above compounds. Only after 5-Aza-dC addition, cells showed an overall increase in fluorescence significant in 1,5-Isoquinolinediol treated cells. This seems to suggest that, once the SDF is inside the cells it doesn't need high activity either of ATM (KU-55933), possibly due to a lack or low levels of double strand breaks in the genome, or of transcription dependent repair pathways (α-Amanitin). The real SFHR-mediated correction efficiency seems to be exactly quantified only after 5-Aza-dC addition, allowing moreover to highlight the effect of 1,5-Isoquinolinediol on overall correction.

The influence that PARP-1 inhibition has on SFHR-modification efficacy could be explained by recent findings reporting how PARP-1 inhibition leads to stalled replication fork with consequent formation of DNA double strand breaks that are resolved by homologous recombination through ATM activation [42]. We believe more in a HR pathway involvement than NHEJ, because our results indicated that SFHR is mainly favored in G_2_/M phase, known to be a phase where this pathway is much more active than in other cell cycle phases. On the other hand it was reported that the inhibition of PARP-1 may increase the methylation of genomic DNA [43], [44]. This may be the reason why a significant increase of fluorescence expression in cells treated with PARP-1 inhibitor, was evident only after 5-Aza-2′-Deoxycytidine addition.

Anyhow a more accessible hypomethylated chromatin may enhance events otherwise not clearly visible. In this regard, a more direct involvement of the PARP-1 repair pathway possibly limiting the efficiency of SFHR may be also proposed; the inhibition of PARP-1 might favor the SDF integration, particularly when the chromatin switches to a hypomethylated open conformation allowing the integration of those residual intracellular SDFs not yet integrated.

Different SFHR-modification efficiencies have been reported [7]–[17], suggesting that the protocol is highly dependent to several experimental conditions, and an exact quantification of gene correction efficiency seems to be crucial to improve the strategy. The assessment and verification of sequence-specific modification of genomic DNA is complicated by the fact that the genomic targets to be modified generally involve endogenous genes that are not readily amenable to enrichment strategies. Therefore a model based on the use of reporter gene and/or selectable marker becomes useful to assess both the overall frequency of targeted DNA repair and to optimize modification protocols.

In this study several evidences about SFHR have been reported: the demonstration of a heritable event of gene modification in a cell clone by molecular analyses, the demonstration of phenotypic reversion (due to genetic modification) by functional assays, and an accurate calculation of gene correction frequency, avoiding artifacts such as those related to PCR-based techniques [45] and to the raising of genomic methylation patterns after correction. Southern blot also demonstrated genomic modification SDF-mediated of the eGFP locus. In addition, site-specific DNA methylation was shown to be quantitatively correlated to the target gene inactivation; these methylation patterns arose with cell replication and appeared to be influenced by the recombination event. Also the involvement of PARP-1-mediated repair system and its interplay with chromatin structure were evidenced. If this involvement is direct or indirect deserves further studies.

By this way additional insight into the comprehension of the parameters mediating the efficacy and specificity of the genomic replacement will be essential to the wider application of these protocols as therapeutic agents.

## Materials and Methods

### Ethic Statement

No ethic statement was required because the study was only performed *in vitro* on an immortalized cell line. Mouse embryonic fibroblast were derived in strict accordance with the recommendations in the Guide for the Care and Use of Laboratory Animals of the National Institutes of Health.

### Cells and culture conditions

Mouse embryonic fibroblasts (MEF) were kindly provided and derived by Dr. Emanuela Bruscia from Yale University (unpublished data) from 13 d.p.c. pregnant female, as described [46]. Isolated cells were cultured in DMEM (Euroclone, Milan, Italy) with 10% FBS (Euroclone, Milan, Italy), 1% L-glutammine (Euroclone, Milan, Italy) and 1% of Penicillin/Streptomycin (Euroclone, Milan, Italy), at 37°C under 5% CO_2_. For cell transformation, SV-40 infection was performed on 1×10^5^ MEF in 4 ml of medium containing 4 µg/ml of polybrene. After the infection, cells were cultured in DMEM, 10% FBS, 1% Non Essential Aminoacids (Invitrogen, CA, USA), 0.01 mM of 2-Beta-Mercaptoethanol (Invitrogen, CA USA), 20 mM Hepes (Euroclone, Milan, Italy), 2 mM L-glutammine (Euroclone, Milan, Italy) and incubated at 37°C under 5% CO_2_. 48 hours after infection cells have been trypsinized and plated with a density of 100 cells/well in a 96-well plate adding 400 µg of G418 (Euroclone, Milan, Italy) for cell selection.

### Construction of the stably integrated mutant and wild-type eGFP cell lines

The wild type eGFP gene was obtained from the pEGFP-N1 vector (Clonetech Lab. Inc., USA) by *XhoI* and *HindIII* restriction (New England Biolabs, Ipswich, MA, USA), and cloned in vector pCR-2.1 (Invitrogen, CA, USA). The wild type sequence of the gene was mutated by QuikChange® Site-Directed Mutagenesis Kit (Agilent Technologies Inc, Santa Clara, CA, USA) using two specific mutagenesis primers (Table 1). The eGFP gene was mutated at codon 70 (**C**AG>**T**AG) creating a stop codon and, at the same time, eliminating a *BtsI* restriction site (in order to be able to screen the corrected clones). The mutated and the wild-type gene were extracted from pCR-2.1 vector by restriction with *XhoI* and *HindIII* and cloned inside the pCEP4 vector (Invitrogen, CA, USA), between pCMV promoter and SV40-pA, using the same restriction enzymes. To create stable cell clones, 3 µg of each *SgrAI* linearized plasmid were added to 1.7×10^6^cells and once transfected cells were plated in 75 cm^2^ flask in fresh medium containing 200 µg/ml of hygromycin (Sigma-Aldrich, Milan, Italy) for stable vector integration selection. The cells were cultured for two weeks in selective medium using 500 µg/ml of hygromycin. After selection, several single cell clones were isolated by serial dilution in 96 well plate and screened by PCR and FISH analyses to check genomic plasmid integration. Among selected clones, D1 was chosen for our experiments, as integrating mut-eGFP gene sequence, and C1 clone as positive control, because containing wt-eGFP gene. Metaphase chromosome preparations for fluorescent in situ hybridization (FISH) were made from D1 and C1 clones. pCEP4was used as probe and slides were processed as previously described [47].

### Plasmid standard curve and copy number determination

To create plasmid standard curve we have followed Applied Biosystems online protocol. For each point of the ten-fold dilution standard curve, the mass of plasmid DNA containing the copies of interest, ranging from 300.000 to 30 copies, has been calculated. qPCR was conducted with 100 ng of DNA in triplicate in a 25 µl reaction using the TaqMan PCR Universal Master Mix and the Applied Biosystems 7500 Fast Real-Time PCR System (Applied Biosystems, Foster City, CA, USA). Primers and probe sequences for eGFP and ApoB (Table 1) were designed using the Primer Express 2.0 software (Applied Biosystems, Foster City, CA, USA). ApoB (endogenous control) was used, in separate reactions, to verify the template DNA concentration and integrity. The final concentration of eGFP/ApoB primers was 400 nM each, and the probe was 150 nM. Reactions followed standard ABI cycling conditions. The copy number of eGFP transgene was calculated as described [48].

### eGFP SDF design, production and methylation

A 876 bp dsDNA SDF homologous to eGFP wild type sequence (named SDF-PCR-WT) was obtained by amplification of the region cloned in pCR-2.1 vector (Invitrogen, CA, USA), using 1f and 1r primers (Fig. 1A, Table 1). The PCR product was purified from 1% agarose gel by QIAquick Gel Extracion Kit (Qiagen, Manchester, U. K). Another kind of ds-DNA SDF (752 bp), homologous to eGFP wild type sequence, was obtained by *HindIII* and *XhoI* restriction of the pCR-2.1 vector (named SDF-DIG-WT). The sequence of the SDFs was checked by DNA sequencing. The ss-SDF-PCR-SDF was obtained by heat denaturation, incubating 10 min at 100°C and soon after placed on ice. All the fragments were dosed by spectrophotometer (ND-1000, Nanodrop, USA). For *in vitro* methylation of SDFs, SDF-PCR-WT was used as target. For *Dam* and *SssI* methylation, 1 µg of SDF-PCR-WT was incubated with 2 units of methyltransferase (New England Biolabs, Ipswich, MA, USA) in a final reaction volume of 20 µl. To create a *Dam/SssI* methylated fragment, we performed a second *SssI* methylation step on previously *Dam* methylated samples. The extent of methylation was checked by incubating overnight treated samples with methylation-sensitive restriction endonucleases followed by PCR amplification (Information S1 and Table 1).

### Cell synchronization

To systematically investigate SFHR-mediated gene repair at various phases of the cell cycle, different concentrations of mimosine, thymidine and vinblastine were tested to synchronize cells in G_0_/G_1_, S or G_2_/M phase, respectively. For the mimosine (Sigma-Aldrich, Milan, Italy) cells were grown for 12 hours at a concentration ranging from 250 µM to 750 µM. For the thymidine (Sigma-Aldrich, Milan, Italy) cells were gown for 15 hours at a concentration ranging from 0.5 mM to 4 mM. Synchronization in G_2_/M phase was obtained growing cells for 14 hours at a concentration ranging from 25 nM to 200 nM of vinblastine (Sigma-Aldrich, Milan, Italy). Once 60% of confluence was reached, cells were treated, washed in PBS, fixed in 70% ethanol, stained with 0.05 mg/ml propidium iodide and then analyzed by FACS-Calibur Flow Cytometer (Becton Dickinson) to determine DNA content. Sync-Wizard Model was used to model the cell cycle. Before any SDFs transfection, for cell cycle phase synchronization, cells were plated at a density of 7×10^5^ in a 150 mm dish, incubated with mimosine for 12 h, or thymidine for 16 h or vinblastine for 14 h.

### Cell transfection and FACS analysis

Electroporation was carried out using the Amaxa Nucleofection System (Lonza, Cologne, Germany). Appropriate Nucleofection program was evaluated in order to have an optimal transfection efficiency and cell viability. We tested two different electroporation programs A-23 and T-20 and two different transfection solutions MEF-1 and MEF-2 using a 21 bp fluorescent oligonucleotide. The combination of program T-20 and solution MEF-2 was chosen (Information S2 and Fig. S2). 12×10^6^ SDFs/cell were added to 1.7×10^6^ synchronized D1 clone cells and suspended in 100 µL of supplemented Nucleofection Buffer MEF-2. Transfection efficiency based on a pMax-GFP (Lonza Cologne, Germany) reporter plasmid was ∼75%. Each time a negative control was used (a mutated 876 bp SDF obtained by PCR). Three days after transfection, cells were FACS analyzed using nucleic acid dye Topro-3 (0.1 µM; Invitrogen, CA, USA) to exclude dead cells. Data from 300.000 live cells were analyzed by the BD-ARIA-DIVA software, to obtain the percentage of eGFP positive cells. To gate eGFP positive cells, parental C1 clone was used. Every experiment was performed in triplicate; every experimental condition was tested in 2 to 3 independent experiments (overall from 6 to 9 independent experimental replicated data).

### KU-55933, 1,5-Isoquinolinediol, α-Amanitin and 5-Aza-2′-Deoxycytidine treatments

Cells were treated by three different inhibitors of specific proteins. KU-55933 (Tocris, Bristol, U.K.), a potent, selective and competitive ATM kinase inhibitor, was used at 10 µM one hour prior transfection. 1,5-Isoquinolinediol (Sigma- Aldrich, Milan, Italy), an inhibitor of Poly-(ADP-ribose) synthetase-1, was used soon after transfection at 0.622 mM for 24 hours.

α-Amanitin (Sigma-Aldrich, Milan, Italy), an inhibitor of eukaryotic RNA polymerase II and III, was used 24 h after transfection at 1 mM for 24 hours. Treated cells, previously synchronized, were then transfected, as described above. To verify a relationship between methylation status of eGFP locus and time- dependent eGFP expression one day after transfection, cells were incubated with 0.5 µM 5-Aza-2′-Deoxycytidine (Sigma-Aldrich, Milan, Italy) for 48 h and then FACS analyzed as described above. Every experiment was performed in triplicate; every experimental condition was tested in 2 to 3 independent experiments (overall from 6 to 9 independent experimental replicated data).

### Southern blot hybridization, sequencing, and restriction analysis

For Southern blot analyses, genomic DNA was isolated from D1 and C1 clones by Flexigene kit (Qiagen, Manchester, U.K.), according to protocol. The genomic DNA was digested (10 µg) by *SalI* and *BtsI*, both cutting inside the pCEP4 vector and analyzed by electrophoresis in 0.8% agarose gel. The DNA was transferred to a nylon Hybond N^+^ membrane (Amersham-Phamacia Biotech, Piscataway,NJ, USA) and hybridized overnight at 65°C with an ^32^P-labeled probe. Probe was generated by PCR from wt-eGFP sequence using the same primer pair for SDF generation. Amplicon was then digested at 37°C with *BtsI* enzyme and the 566 bp band was gel purified and used as probe. Successful site-specific T to C conversion in the eGFP reporter gene was evidenced by: i) sequence analysis; ii) restriction enzyme analysis and iii) allelic discrimination (Information S1). For sequencing and restriction analyses, genomic DNA was isolated from transfected, untransfected and sorted corrected cells, as described above, and eGFP locus amplified by using RFLP-f and RFLP-r primers (Fig. 4B, Table 1) located outside the SDF region. The PCR product (986 bp) was resolved by 1% agarose gel electrophoresis, purified using QIAquick gel extraction kit (Qiagen, Manchester, U.K.) and resuspended in demineralized water. Gel purified amplicons were directly sequenced by cycle sequencing with a BigDye Terminator v3.1 Cycle Sequencing Kit (Applied Biosystems, Foster City, CA, USA) using the same primer pair as for amplification. The sequencing reactions were carried out in a 10 µl final volume according to manufacture protocol. Electrophoretic separation was carried out on an ABI Prism 310 Genetic Analyzer (Applied Biosystems, Foster City, CA, USA). To asses gene modification, a *BtsI* restriction digestion at 55°C was carried on gel purified amplicons. Products were analyzed on a 2% agarose gel.

### RNA analysis of eGFP gene

C1 and D1 fluorescent cells were plated after sorting in a 100 mm plate 24 h prior to 0.5 µM 5-Aza-2′-Deoxycytidine (Sigma-Aldrich, Milan, Italy) treatment and incubated for 48 h. After treatment, cells were analyzed for eGFP expression. Total RNA was extracted according to Trizol protocol (Invitrogen, CA, USA) and 1.5 µg of RNA were reverse transcribed to cDNA according High-Capacity cDNA Archive kit protocol (Applied Biosystems, Foster City, CA). Real time RT-PCR was performed using a 7500 Fast Real-Time PCR System (Applied Biosystems, Foster City, CA, USA) using the same primers for copy number determination (Table 1). A commercially available endogenous gene, Glyceraldehyde 3-phosphate dehydrogenase gene (GAPDH: Mm99999915g1 Applied Biosystems) was used as reference for the TaqMan assay. A comparative Ct method was used to quantify relative gene expression. All PCR reactions were performed in triplicate.

### DNA methylation analysis

In order to characterize the relationship between methylation status of eGFP locus and time-dependent eGFP expression, studies by multiplex *HpaII*/PCR or *AciI*/PCR were performed on genomic DNA of C1 and D1 modified clones, treated by *HpaII* or *AciI* methylation-sensitive restriction endonucleases (New England Biolabs, Ipswich, MA, USA). The methylation status of different sites of the locus were analyzed by the selection of 3 regions: the upstream/SFHR target region (indicated as c amplicon, Fig. 7A), the SFHR target/downstream region (indicated as d amplicon; Fig. 7A) and the downstream region (indicated as e amplicon; Fig. 7A). Two regions which possesses no *HpaII* or *AciI* recognition sites, were used as internal standards for *HpaII*/PCR and *AciI*/PCR (indicated respectively as St3 and St4 amplicon, Fig. 7A). Primer pairs were designed (Table 1) with at least one primer located outside the SFHR targeted region avoiding the amplification of non integrated SDF. 300 ng of genomic DNA were digested at 37°C with 3 units of each enzyme for 12 hours in a final volume of 20 µl. Only after the treatment with methylation-sensitive restriction endonucleases, that cut the target genomic DNA only if unmethylated, every single region was amplified together with the internal standard in a multiplex touchdown PCR. The touchdown PCR cycle was performed as follow using a PTC100 Thermal Cycler (Bio- Rad): 2′ of at 92°C, 40 cycles (45″ at 94°C, 1′30″ at 64°C −0.2°C per cycle, and 4′ at 72°C) and a final extension of 7′ at 72°C. Gel electrophoresis run was scanned by a CCD camera (VisiDoc-It, UVP) and acquired on the VisionWorks LS software version 6.7.3 (UVP) for densitometry. For a semi-quantitative evaluation of methylation patterns of integrated eGFP construct, each target amplicon was normalized for the corresponding control amplicon. The final result is the percentage of methylation of the examined region respect to corresponding uncut controls. All PCR reactions ad densitometric analyses were performed at least in triplicate.

### Statistical analysis

All data were compared by using the two-tailed, paired Student's t test analysis. Methylation densitometry data were analyzed using analysis of variance (ANOVA). A p<0.05 was considered statistically significant.

## Supporting Information

Figure S1 **Fluorescent in situ hybridization (FISH) on D1 clone.** The FISH analysis shows chromosomal localization of the transgene. Arrows indicate the hybridization signals.
(TIF)

Figure S2 **Optimization of nucleofection protocol by FACS analysis after transfecting a 21 bp fluorescent oligonucleotide.** A) A representative density plot in which R1 identifies the viable D1 cell population. Dead cells were excluded by propidium iodide staining. Histograms of R1-gated D1 cells are shown. In the first row cells transfected by A-23 and T-20 programs are analyzed immediately after oligonucleotide transfection (T = 0 h). In the second row the same analysis was performed 24 hours after transfection (T = 24 h). Control (Ctr) was transfected with a non fluorescent oligonucleotide. Fluorescence intensity was measured on the X-axis at 530±30 nm wavelength. The Mean Fluorescence Intensity (MFI) values showed in the histograms suggested a transfection efficiency similar for A-23 and T-20 programs at T = 0 h. A decrease of fluorescence was detected after 24 hours in A-23 transfected cells respect to T-20 ones. B) Comparison of viability and transfection efficiency testing T-20 and A-23 programs with two distinct transfection solutions (MEF-1 and MEF-2). Combination of T-20 program and MEF-2 solution gave the highest transfection efficiency together with low cell death.
(TIF)

Figure S3 **Representative dot plots of D1 modification efficiencies obtained testing different amount of SDF.** A SDF homologous to mutated eGFP sequence was used as control (CTR). See Fig. 2A for overall results.
(TIF)

Figure S4 **Representative dot plots of D1 modification efficiencies obtained testing SDFs synthesized with different experimental protocols..** A SDF homologous to mutated eGFP sequence was used as control (CTR). See Fig. 2B for overall results.
(TIF)

Figure S5 **Representative dot plots of D1 modification efficiencies after cell cycle synchronization.** A SDF homologous to mutated eGFP sequence was used as control (CTR). See Fig. 3B for overall results.
(TIF)

Figure S6 **Representative dot plots of D1 modification efficiencies obtained testing several differently methylated SDFs.** See Fig. 3C for overall results.
(TIF)

Figure S7 **Analysis of methylation patterns of SDF-PCR-WT treated with DNA-methyltransferases and of SDF-DIG-WT.** A) Analysis design. B) SDF-PCR-WT and SDF-DIG-WT were treated by methylation sensitive restriction enzymes for *HpaII*, *Dcm* or *Dam* methylation and used as target for following amplification (central panels). Untreated samples, or samples treated with heat inactivated restriction enzymes (left panels) resulted to be uncut. Samples cut with methylation insensitive isoschizomers (right panels: *MspI, BstNI, Sau3AI*) are shown as controls. In all panels the lower band is the internal control of amplification (amplicon from a zone without recognition sites for restriction enzymes) always amplified, while the upper band is the amplicon from the target sequence (see Fig. S7A for the description of zones). The recognition sequence of each restriction enzyme is reported on the right; the nucleotide that, if methylated, prevents the cut by the sensitive restriction enzyme is underlined. In every lane M a GeneRuler™ 50 bp DNA Ladder is shown. For *HpaII* and *Dcm* methylations target samples are repeated in the same order, as follows: lanes 1 and 2 SDF-DIG-WT; lane 3, SDF-PCR-WT; lane 4, pCR 2.1 plasmid; lane 5, negative control (with water instead of DNA). For *Dam* methylation: lane 1, SDF-PCR-WT; lane 2, pCR2.1 plasmid; lane 3 negative control. C) Amplicons obtained from PCR-amplified (primers 1F/1R) SDF *in vitro* methylated by *Dam* methyltransferase and *MboI* treated (lane 1), methylated by *SssI* methyltransferase and *HpaII* treated (lane 3) as well as methylated by both *Dam* and *SssI* and treated by both *MboI* and *HpaII* (lane 5) are shown. Respective negative controls, using unmethylated SDF as target (lanes 2, 4, 6) are shown. Lanes M represents GeneRuler™ 50 bp DNA Ladder. The methylating treatment resulted effective, as superimposed methylation pattern protect SDF from digestion. These methylated SDF are those used to test the effect of SDF methylation on correction efficiency.
(TIF)

Figure S8 **Allelic discrimination plot.** Red and blue dots represent wild-type (D1 sorted positive and parental C1 clone) and mutated (D1-CTR) genotype, respectively.
(TIF)

Figure S9 **CpG Methylation analysis of** ***HpaII*** **sites in C1 and D1 clones.** A) Experimental design. For both B and C panels the treatment of genomic DNA with active or non active *HpaII* is indicated; in each panel, the upper band is the analyzed zone (c, d, e amplicons) while the lower band is the control amplicon (St3); B) eGFP negative (lanes 1–3), more positive (lanes 4–6), less positive (lanes 7–9) C1 parental cells; M: 50 bp marker. PCR blanks corresponds to negative controls (no DNA; lanes 10 and 11). C) D1 eGFP negative (lanes 1–3), and positive (lanes 4–6) cells; M: 50 bp marker. PCR blanks corresponds to negative controls (no DNA; lanes 7 and 8).
(TIF)

Figure S10 **CpG Methylation analysis of** ***AciI*** **sites in C1 and D1 clones.** A) Experimental design. For both B and C panels the treatment of genomic DNA with active or non active *Aci I* is indicated; in each panel, the upper band is the analyzed zone (c, d, e amplicons) while the lower band is the control amplicon (St4); B) eGFP negative (lanes 1–3), more positive (lanes 4–6), less positive (lanes 7–9) C1 cells; M: 50 bp marker. PCR blanks corresponds to negative controls (no DNA; lanes 10 and 11). C) D1 eGFP negative (lanes 1–3), and positive (lanes 4–6) cells; M: 50 bp marker. PCR blanks corresponds to negative controls (no DNA; lane 7 and 8).
(TIF)

Information S1 **Supporting Materials and Methods.**
(DOC)

Information S2 **Supporting Results.**
(DOC)
