# Supplementary figures and images for: Small Fragment Homologous Replacement: Evaluation of Factors Influencing Modification Efficiency in an Eukaryotic Assay System
Source: PLoS One. 2012 Feb 16;7(2):e30851. doi: 10.1371/journal.pone.0030851 (PMC3281040; doi:10.1371/journal.pone.0030851)

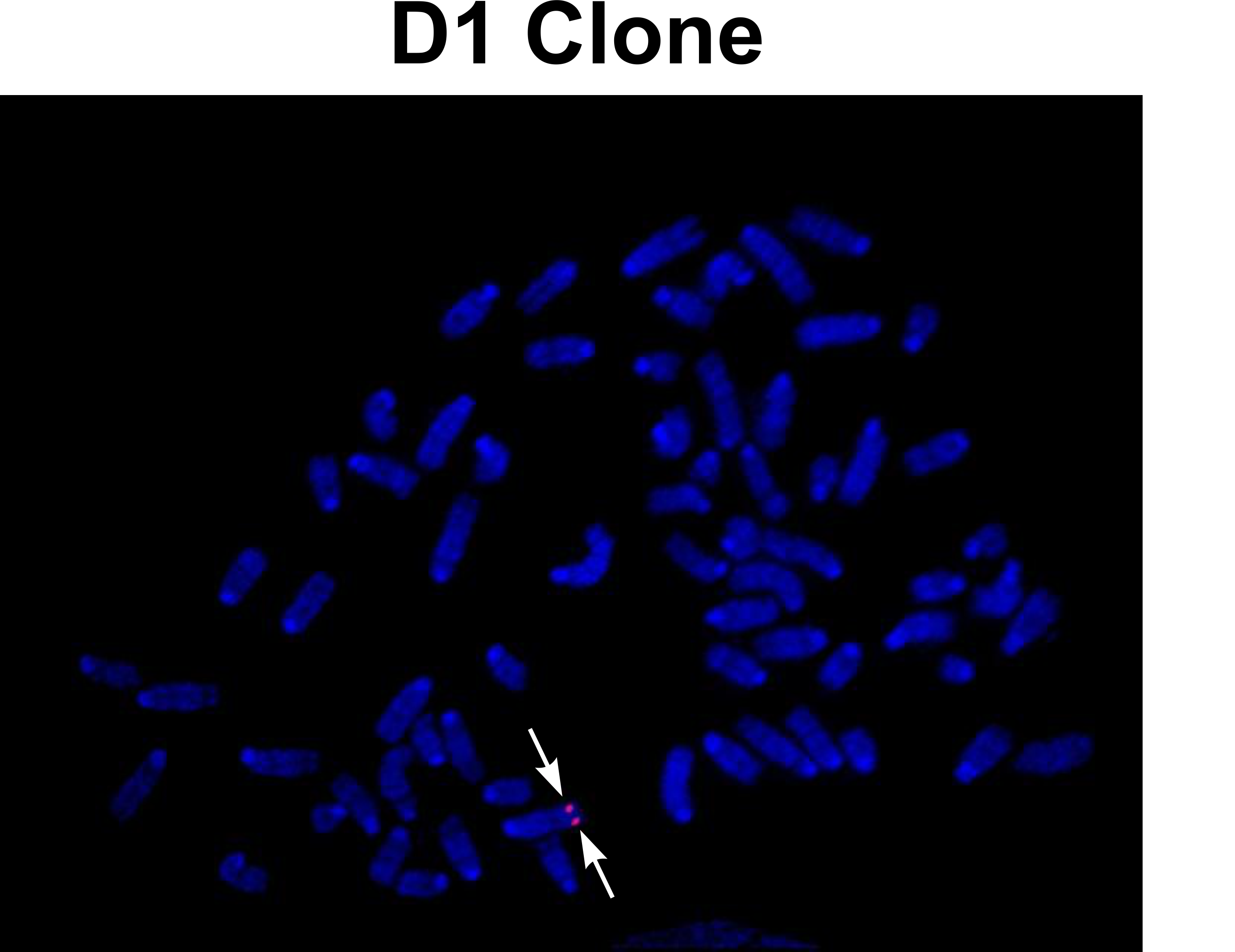

Supplement: Figure S1 — Fluorescent in situ hybridization (FISH) on D1 clone. The FISH analysis shows chromosomal localization of the transgene. Arrows indicate the hybridization signals. (TIF) [file pone.0030851.s001.tif]

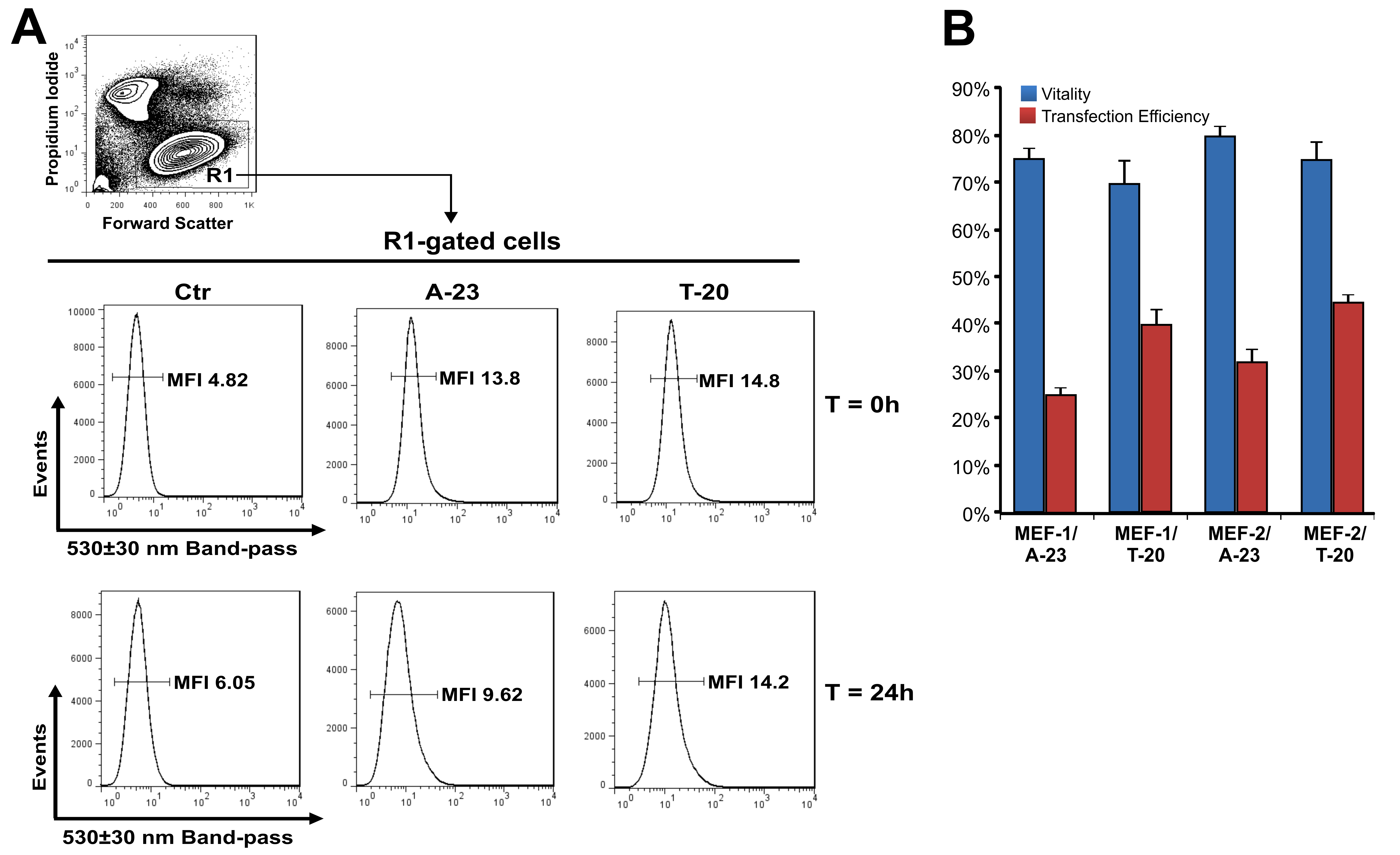

Supplement: Figure S2 — Optimization of nucleofection protocol by FACS analysis after transfecting a 21 bp fluorescent oligonucleotide. A) A representative density plot in which R1 identifies the viable D1 cell population. Dead cells were excluded by propidium iodide staining. Histograms of R1-gated D1 cells are shown. In the first row cells transfected by A-23 and T-20 programs are analyzed immediately after oligonucleotide transfection (T = 0 h). In the second row the same analysis was performed 24 hours after transfection (T = 24 h). Control (Ctr) was transfected with a non fluorescent oligonucleotide. Fluorescence intensity was measured on the X-axis at 530±30 nm wavelength. The Mean Fluorescence Intensity (MFI) values showed in the histograms suggested a transfection efficiency similar for A-23 and T-20 programs at T = 0 h. A decrease of fluorescence was detected after 24 hours in A-23 transfected cells respect to T-20 ones. B) Comparison of viability and transfection efficiency testing T-20 and A-23 programs with two distinct transfection solutions (MEF-1 and MEF-2). Combination of T-20 program and MEF-2 solution gave the highest transfection efficiency together with low cell death. (TIF) [file pone.0030851.s002.tif]

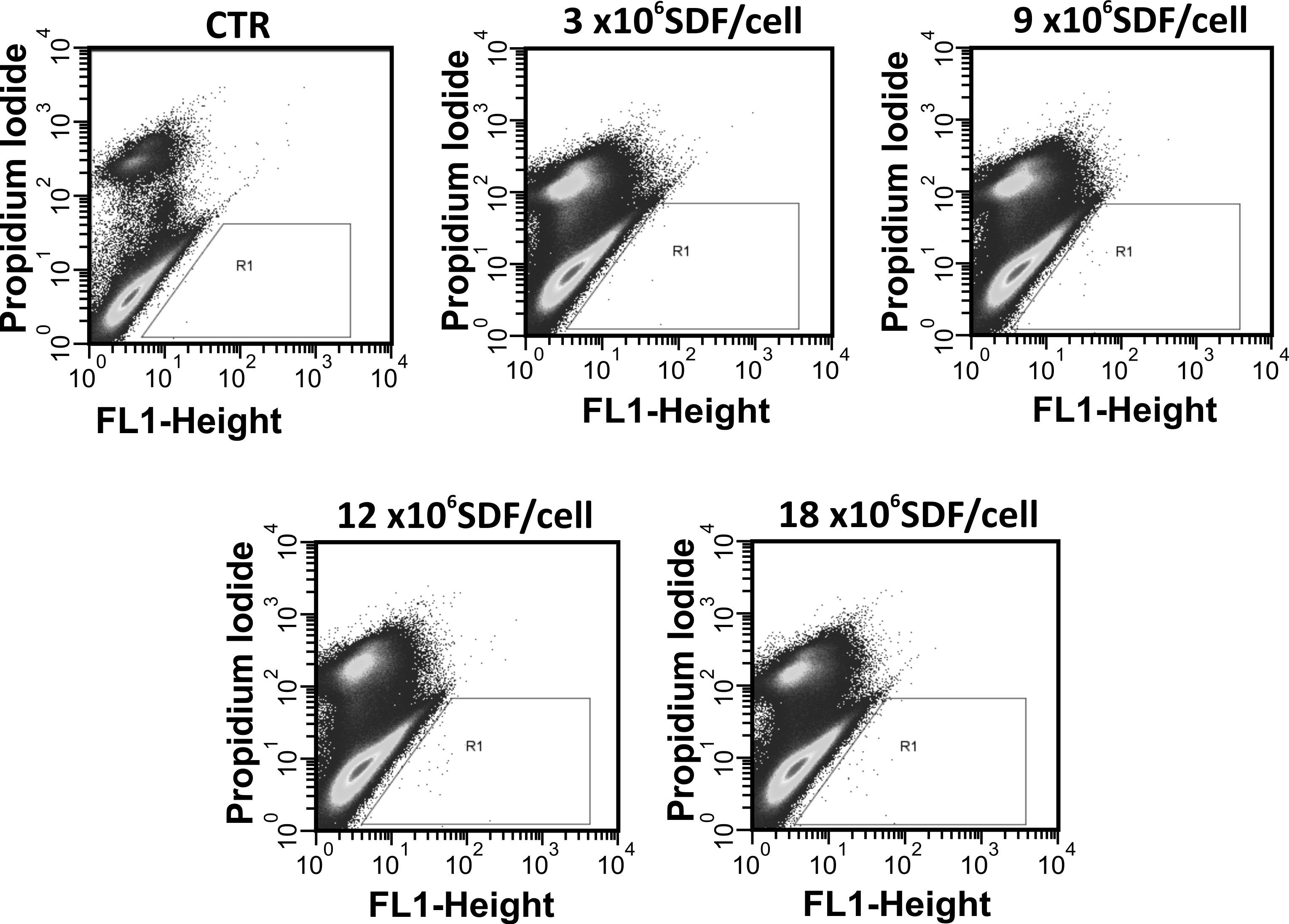

Supplement: Figure S3 — Representative dot plots of D1 modification efficiencies obtained testing different amount of SDF. A SDF homologous to mutated eGFP sequence was used as control (CTR). See Fig. 2A for overall results. (TIF) [file pone.0030851.s003.tif]

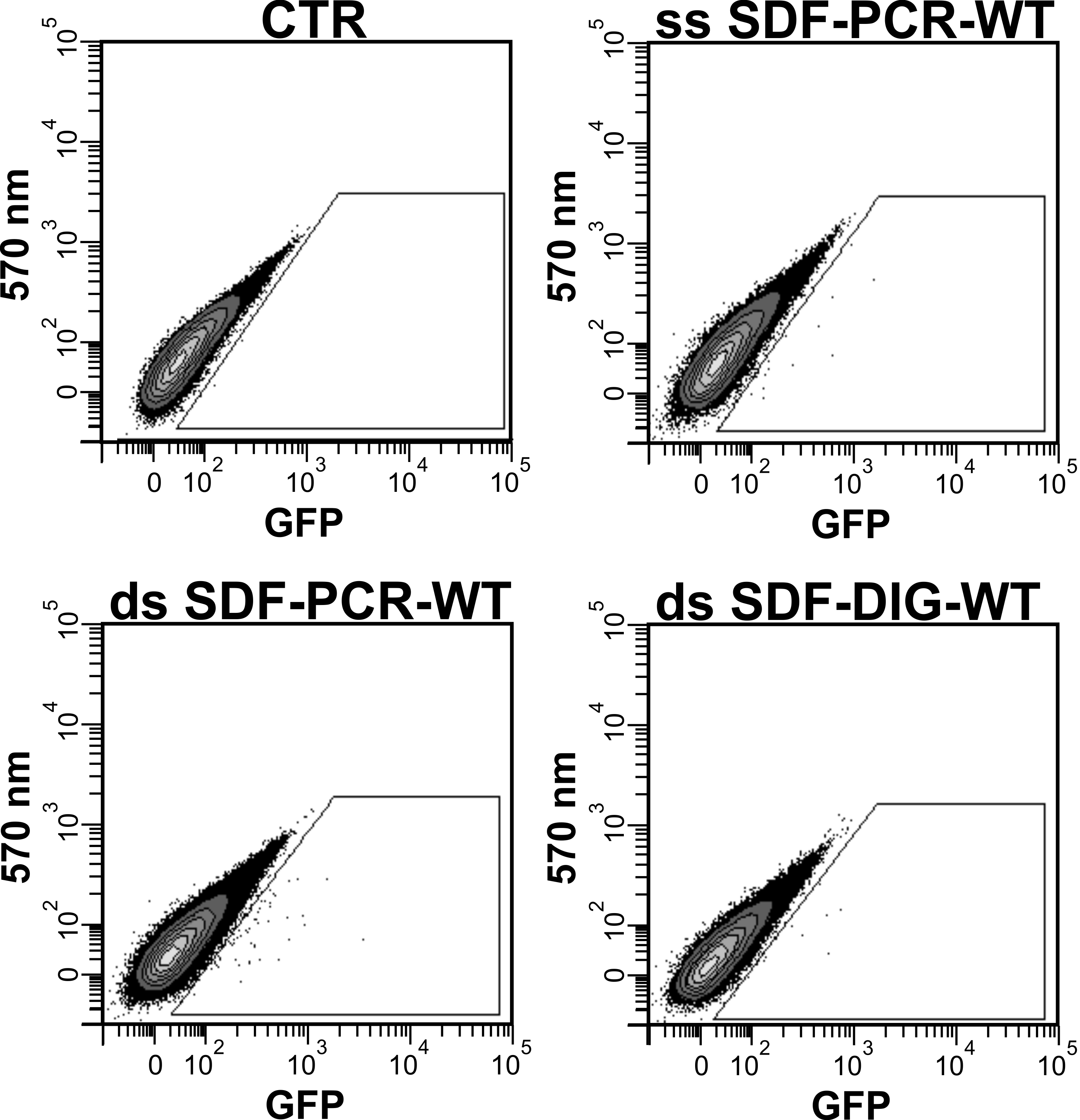

Supplement: Figure S4 — Representative dot plots of D1 modification efficiencies obtained testing SDFs synthesized with different experimental protocols.. A SDF homologous to mutated eGFP sequence was used as control (CTR). See Fig. 2B for overall results. (TIF) [file pone.0030851.s004.tif]

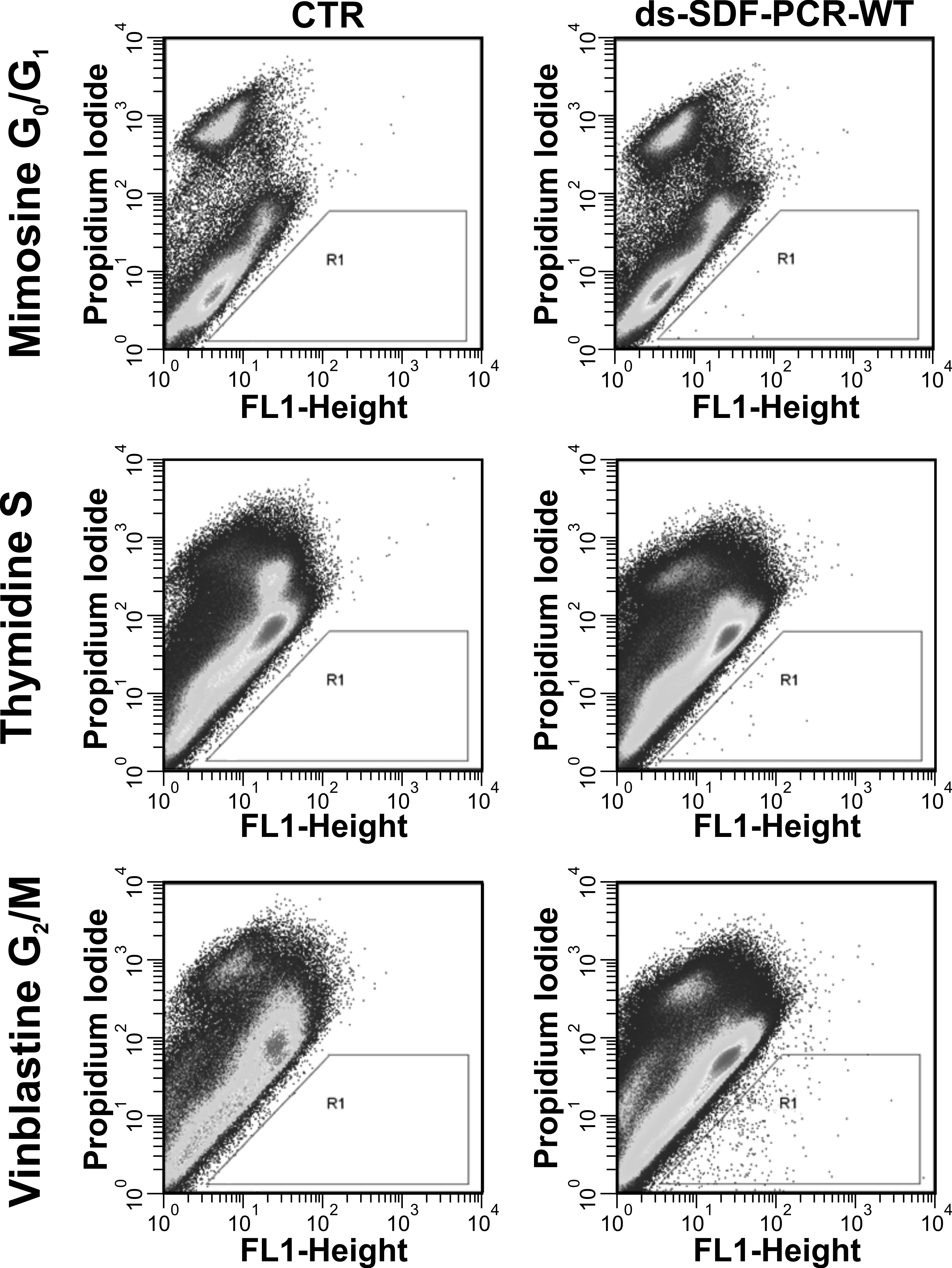

Supplement: Figure S5 — Representative dot plots of D1 modification efficiencies after cell cycle synchronization. A SDF homologous to mutated eGFP sequence was used as control (CTR). See Fig. 3B for overall results. (TIF) [file pone.0030851.s005.tif]

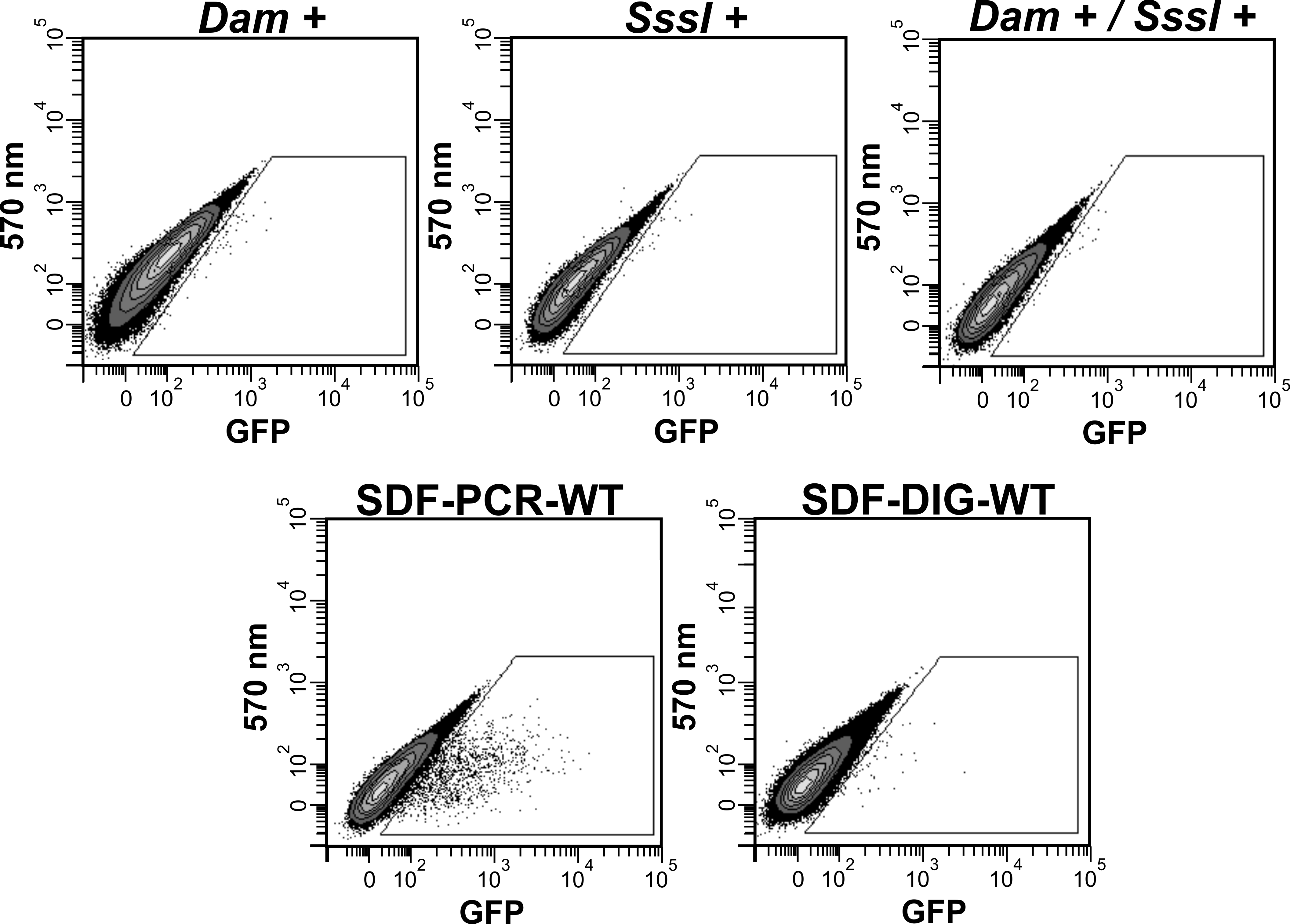

Supplement: Figure S6 — Representative dot plots of D1 modification efficiencies obtained testing several differently methylated SDFs. See Fig. 3C for overall results. (TIF) [file pone.0030851.s006.tif]

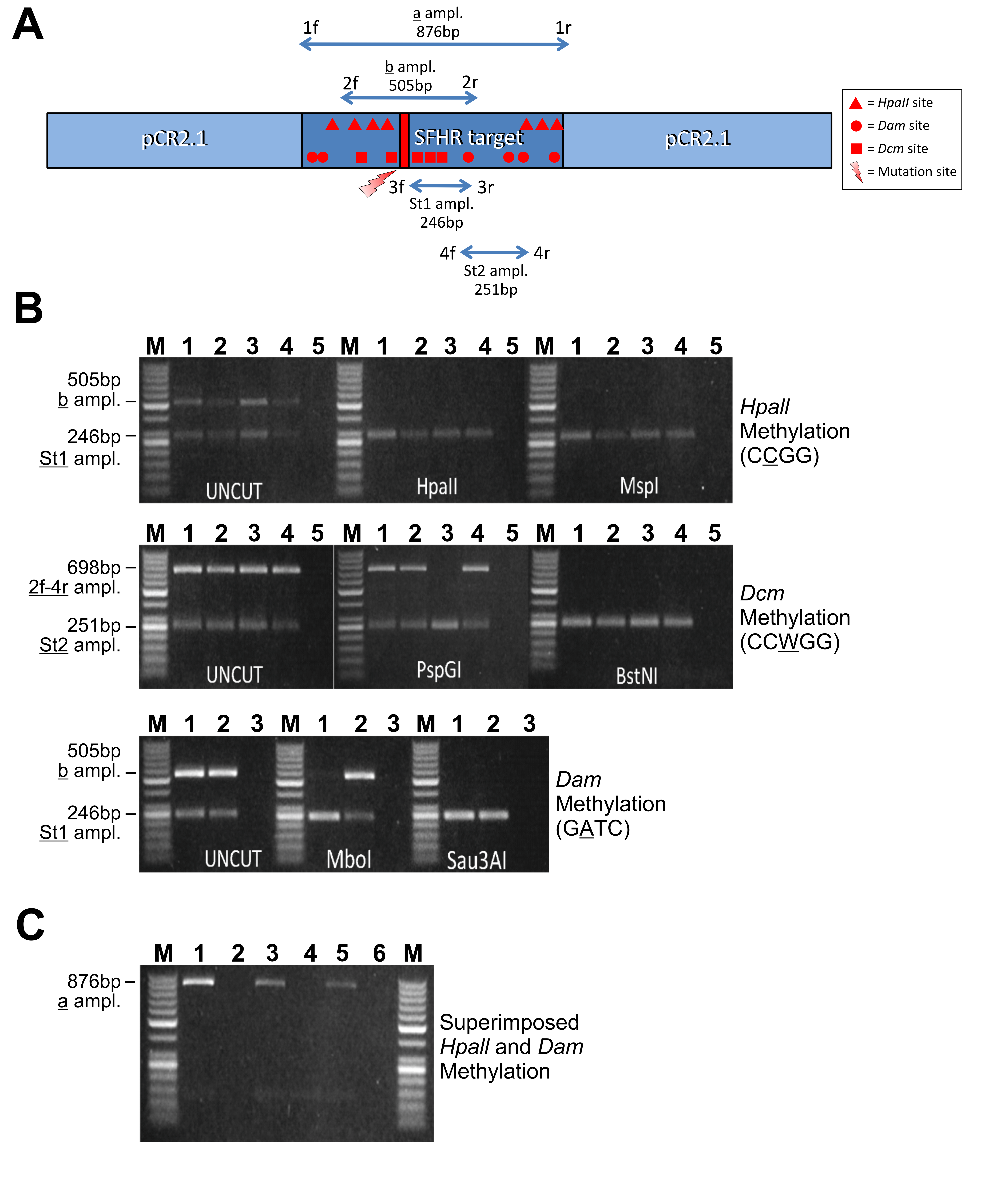

Supplement: Figure S7 — Analysis of methylation patterns of SDF-PCR-WT treated with DNA-methyltransferases and of SDF-DIG-WT. A) Analysis design. B) SDF-PCR-WT and SDF-DIG-WT were treated by methylation sensitive restriction enzymes for HpaII, Dcm or Dam methylation and used as target for following amplification (central panels). Untreated samples, or samples treated with heat inactivated restriction enzymes (left panels) resulted to be uncut. Samples cut with methylation insensitive isoschizomers (right panels: MspI, BstNI, Sau3AI) are shown as controls. In all panels the lower band is the internal control of amplification (amplicon from a zone without recognition sites for restriction enzymes) always amplified, while the upper band is the amplicon from the target sequence (see Fig. S7A for the description of zones). The recognition sequence of each restriction enzyme is reported on the right; the nucleotide that, if methylated, prevents the cut by the sensitive restriction enzyme is underlined. In every lane M a GeneRuler™ 50 bp DNA Ladder is shown. For HpaII and Dcm methylations target samples are repeated in the same order, as follows: lanes 1 and 2 SDF-DIG-WT; lane 3, SDF-PCR-WT; lane 4, pCR 2.1 plasmid; lane 5, negative control (with water instead of DNA). For Dam methylation: lane 1, SDF-PCR-WT; lane 2, pCR2.1 plasmid; lane 3 negative control. C) Amplicons obtained from PCR-amplified (primers 1F/1R) SDF in vitro methylated by Dam methyltransferase and MboI treated (lane 1), methylated by SssI methyltransferase and HpaII treated (lane 3) as well as methylated by both Dam and SssI and treated by both MboI and HpaII (lane 5) are shown. Respective negative controls, using unmethylated SDF as target (lanes 2, 4, 6) are shown. Lanes M represents GeneRuler™ 50 bp DNA Ladder. The methylating treatment resulted effective, as superimposed methylation pattern protect SDF from digestion. These methylated SDF are those used to test the effect of SDF methylation on correction efficiency. (TIF) [file pone.0030851.s007.tif]

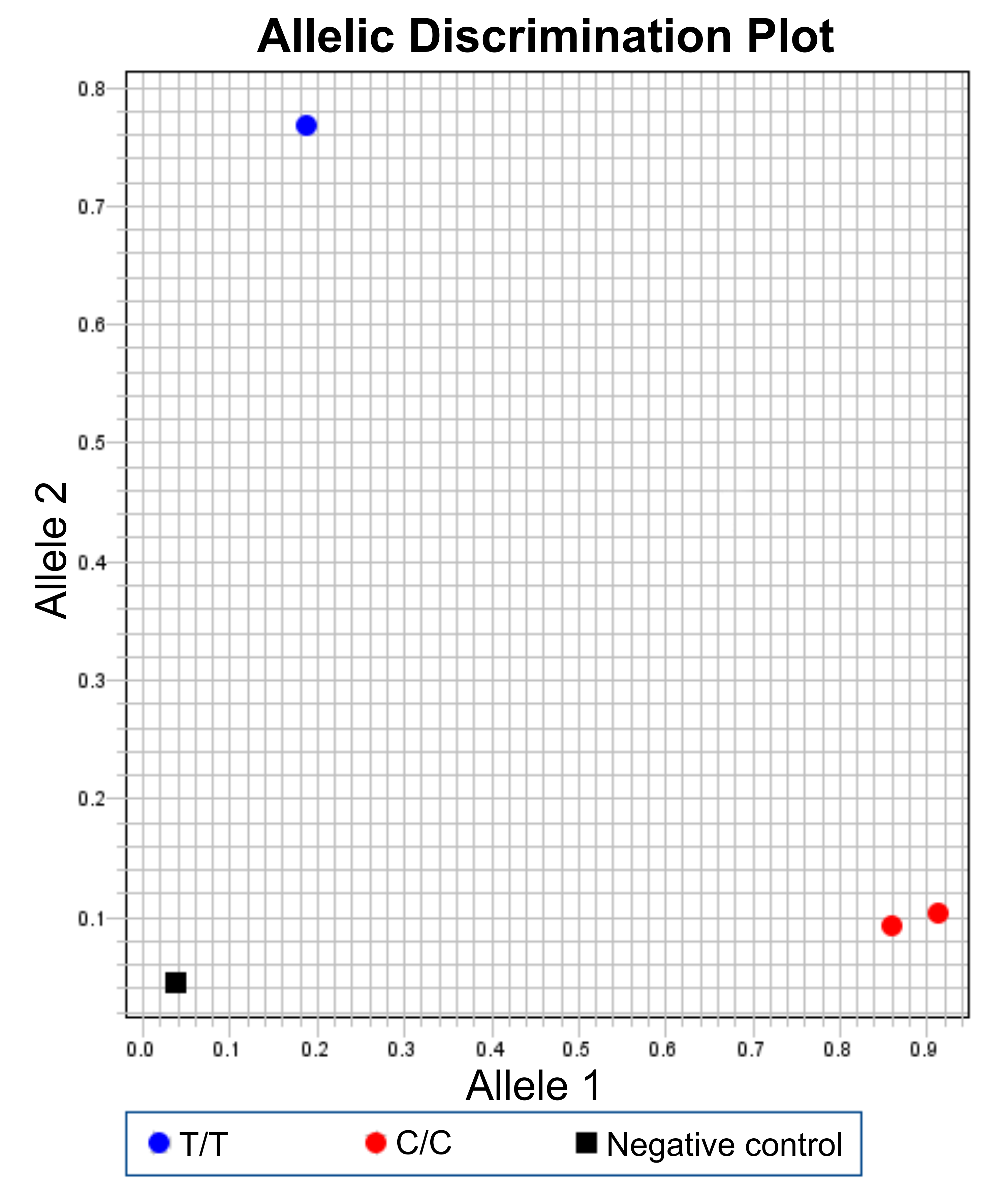

Supplement: Figure S8 — Allelic discrimination plot. Red and blue dots represent wild-type (D1 sorted positive and parental C1 clone) and mutated (D1-CTR) genotype, respectively. (TIF) [file pone.0030851.s008.tif]

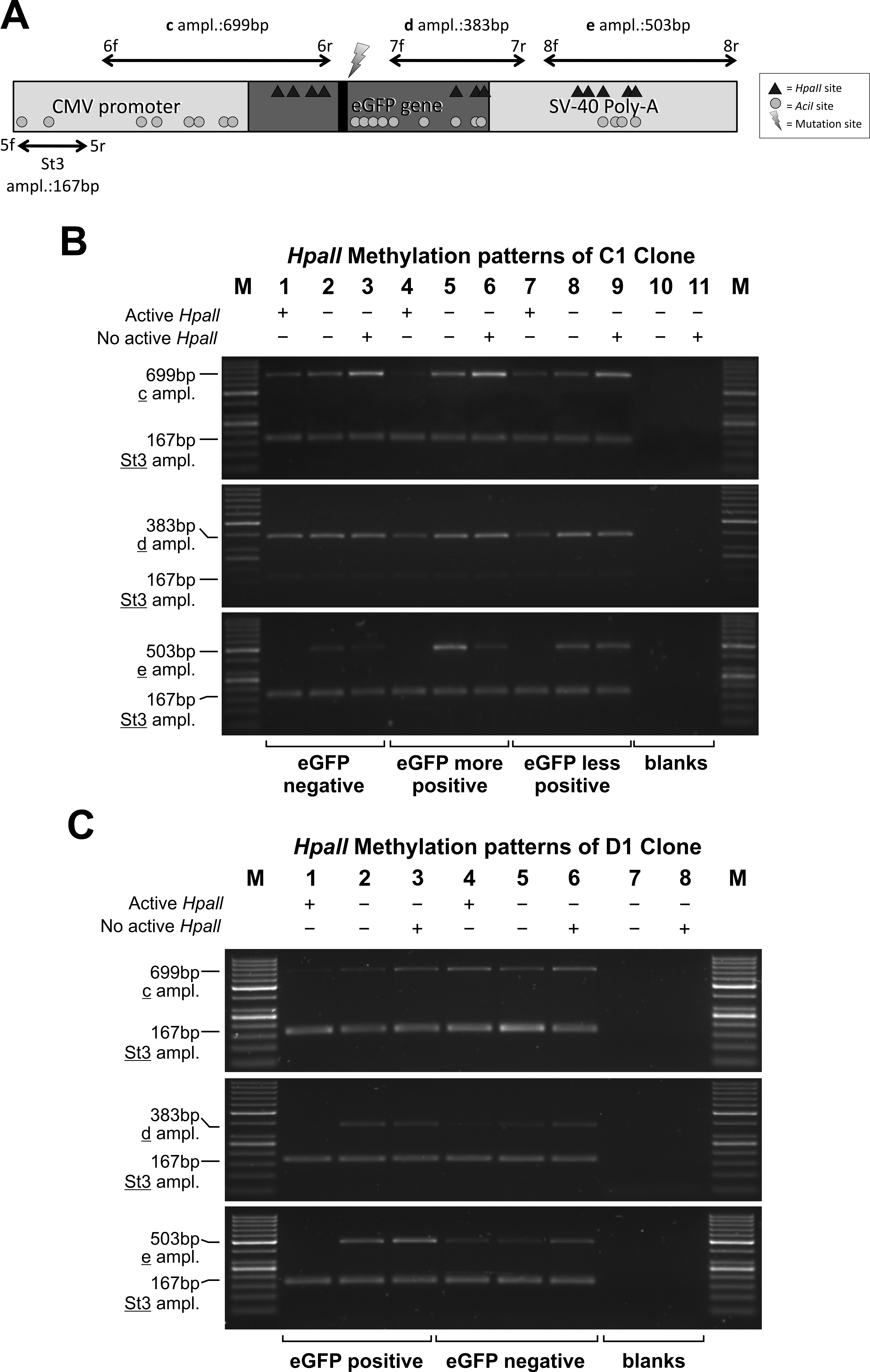

Supplement: Figure S9 — CpG Methylation analysis of HpaII sites in C1 and D1 clones. A) Experimental design. For both B and C panels the treatment of genomic DNA with active or non active HpaII is indicated; in each panel, the upper band is the analyzed zone (c, d, e amplicons) while the lower band is the control amplicon (St3); B) eGFP negative (lanes 1–3), more positive (lanes 4–6), less positive (lanes 7–9) C1 parental cells; M: 50 bp marker. PCR blanks corresponds to negative controls (no DNA; lanes 10 and 11). C) D1 eGFP negative (lanes 1–3), and positive (lanes 4–6) cells; M: 50 bp marker. PCR blanks corresponds to negative controls (no DNA; lanes 7 and 8). (TIF) [file pone.0030851.s009.tif]

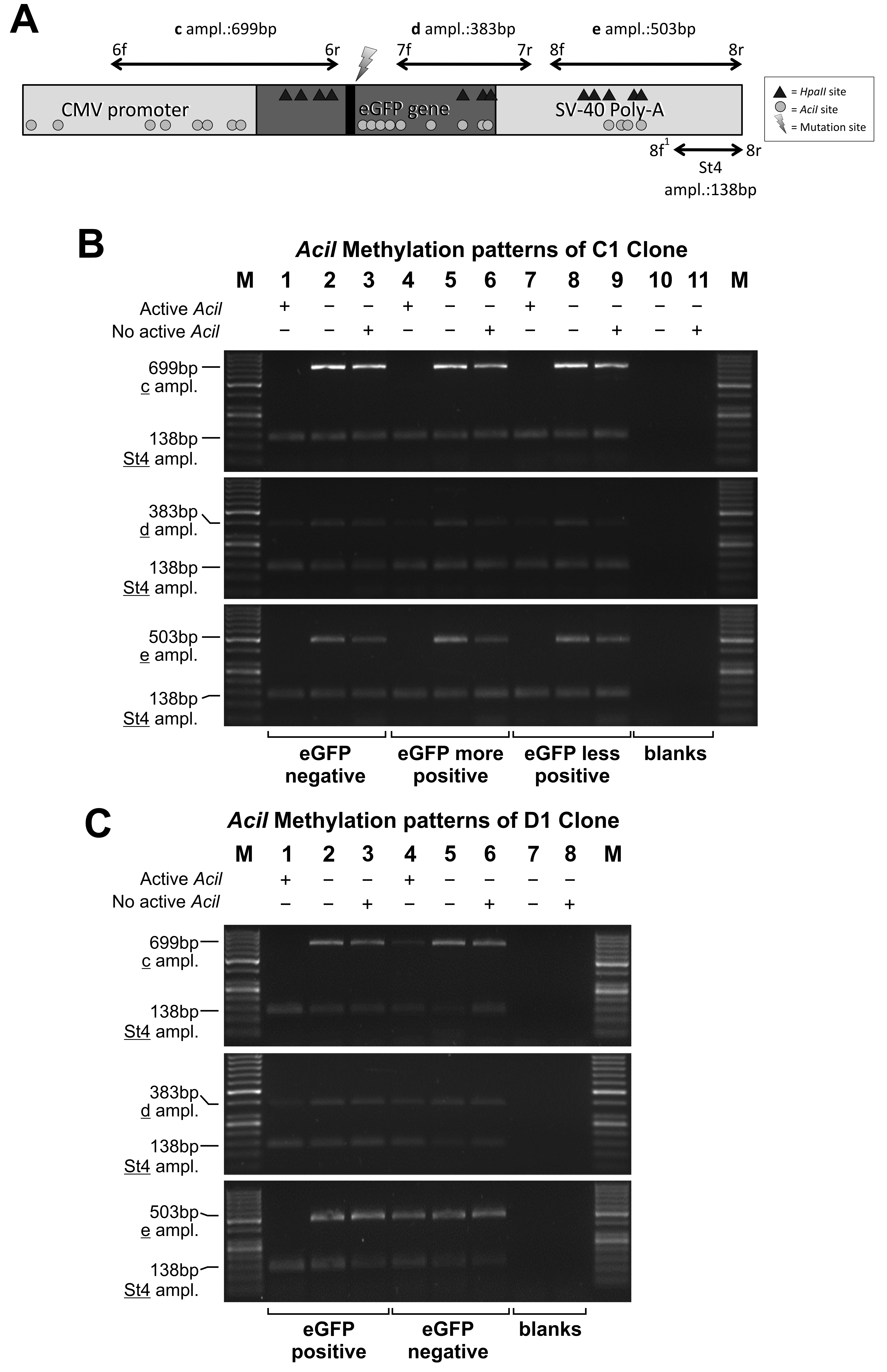

Supplement: Figure S10 — CpG Methylation analysis of AciI sites in C1 and D1 clones. A) Experimental design. For both B and C panels the treatment of genomic DNA with active or non active Aci I is indicated; in each panel, the upper band is the analyzed zone (c, d, e amplicons) while the lower band is the control amplicon (St4); B) eGFP negative (lanes 1–3), more positive (lanes 4–6), less positive (lanes 7–9) C1 cells; M: 50 bp marker. PCR blanks corresponds to negative controls (no DNA; lanes 10 and 11). C) D1 eGFP negative (lanes 1–3), and positive (lanes 4–6) cells; M: 50 bp marker. PCR blanks corresponds to negative controls (no DNA; lane 7 and 8). (TIF) [file pone.0030851.s010.tif]
