# Supplementary material for: Small Fragment Homologous Replacement: Evaluation of Factors Influencing Modification Efficiency in an Eukaryotic Assay System
Source: PLoS One. 2012 Feb 16;7(2):e30851. doi: 10.1371/journal.pone.0030851 (PMC3281040; doi:10.1371/journal.pone.0030851)
Supplement: Information S1 — Supporting Materials and Methods. (DOC) [file pone.0030851.s011.doc]

**SUPPORTING INFORMATION 1**

**Supporting Materials and Methods**

**Methylation analyses of SDF.**

The methylation patterns of SDF-DIG-WT and SDF-PCR-WT (Fig. S7B), as well as of SDF-PCR-WT with superimposed methylation (Fig. S7C) were tested by treating the targets with specific metyhylation-sensitive (eukaryotic, Dcm, Dam) restriction endonucleases *(HpaII or MspI*, *PspGI* or *BstNI*, *MboI* and *Sau3AI*). PCR amplification with flanking primers was performed after restriction (Table 1). Specifically, 300 ng of DNA were digested at 37 °C with 3 units of enzyme for 12 hours in a total volume of 20 µl. PCR was performed in a volume of 15 μl containing: 6 pmol of each primer, 6 ng of genomic DNA, 175 μM dNTPs, 0.5 unit of Yieldace DNA Polymerase (Agilent Technologies), and 1X Yieldace reaction buffer. The reaction was a multiplex PCR of both the specific target and the internal standard (Table 1). PCR cycle was 2’ at 92 °C, 45’’ at 94 °C, 1’30’’ at specific Ta (Table 1) and 2’30” at 72 °C for 20 cycles.

**Allelic Discrimination protocol.**

Genotyping analysis was carried out on purified PCR (RFLP Amplicon) on a 7500 Fast Real-Time PCR System using a TaqMan MGB Custom Probe able to discriminate between wild type and mutant alleles. The reaction mixture for the assay was as follows: 6.25 μl 1× TaqMan® Universal Master Mix, 0.25 μl TaqMan Genotyping Assay and water to a final volume of 12.5 μl. Cycle conditions were 50°C for 2 min, 95°C for 10 min, 35 cycles of 95°C for 30 s and 62°C for 1 min, performed in a 96-well optical plate. Each plate contained positive control and a negative control. Genotypes were manually scored using Sequence Detection Software 2.0 (Applied Biosystems).
