# Supplementary material for: Small Fragment Homologous Replacement: Evaluation of Factors Influencing Modification Efficiency in an Eukaryotic Assay System
Source: PLoS One. 2012 Feb 16;7(2):e30851. doi: 10.1371/journal.pone.0030851 (PMC3281040; doi:10.1371/journal.pone.0030851)
Supplement: Information S2 — Supporting Results. (DOC) [file pone.0030851.s012.doc]

**SUPPORTING INFORMATION 2**

**Supporting Results**

**Transfection parameters setting.** To identify the program and the solution with the highest transfection efficiency and, at the same time, the lowest mortality, we transfected a labelled oligonucleotide, 21 bp long, testing two different programs (T-20 and A-23) combined with two different solutions (MEF-1 or MEF-2). Oligonucleotide-related fluorescence and survival in treated cells were determined by FACS analysis at time zero (T=0h) and 24 hours after (T=24h) transfection, comparing them to control samples (CTR) in which no-labelled oligonucleotide was transfected (Fig. S2A). Data obtained indicated T-20 as best program when used with MEF-2 solution (Fig. S2B).

**Analysis of methylation patterns of pCR 2.1 plasmid and of SDF-DIG-WT**

To clarify the molecular mechanism responsible of a lower correction efficiency of SDF-DIG-WT respect to SDF-PCR-WT, the methylation patterns of SDF-DIG-WT, SDF-PCR-WT and of pCR 2.1 plasmid containing SDF and used as control were studied (Fig. S7B). These samples were treated with methylation-sensitive or insensitive restriction endonucleases, and used as targets for subsequent PCR amplification. For each sample, a multiplex PCR with two amplicons was obtained: the smallest is referred to a zone with no restriction sites (the internal standard always amplified), the largest is the amplicon of the target zone. Both *Dcm* and *Dam* bacterial methylation patterns resulted present on pCR2.1 plasmid and on the SDF-DIG-WT, as clearly evidenced by the presence of an amplified largest band in samples treated by the methylation sensitive *PspGI* and *MboI*, respectively. There is no eukaryotic *HpaII* methylation pattern on pCR2.1 and on SDF-DIG-WT, as demonstrated by the absence of the specific amplicon in *HpaII*-treated samples. As expected,SDF-PCR-WT showed no methylation pattern, as evidenced by the absence of any specific amplicon from *HpaII*-, *PspGI*- or *MboI*-treated samples.

**Confirmation of superimposed methylation patterns after treatment of SDF-PCR-WT with DNA- methyltransferases**

An unmethylated PCR-amplified SDF underwent to methylating treatment by specific *SssI*, *Dam*, and both DNA methyltransferases (Materials and Methods in the mail paper). To check for the successful methylation, these SDFs underwent to digestion with the respective methylation-sensitive restriction endonucleases and were used as target for following PCR amplification (Fig. S7C). The successful amplification of the target previously treated with a DNA-methyltransferase and the respective methylation sensitive restriction endonuclease, demonstrates the presence of the specific methylation pattern.

**Analysis of *HpaII* methylation patterns of integrated eGFP in C1 and D1 clone.** From *HpaII* methylation pattern analysis (Fig. S9B and Fig. 7B), the d amplicon resulted more methylated than c amplicon in both the non fluorescent (lane 1) and fluorescent (lanes 4 and 7) parental C1 cells. The e amplicon resulted always unmethylated (lanes 1, 4, 7). In both c and d amplicons, the methylation level is proportional to eGFP fluorescence. SDF-modified and re-sorted fluorescent D1 clone (Fig. S9C and 7C) generally showed no or very low methylation (lane 1), while non fluorescent cells (lane 4) showed very high levels of methylation.

**Analysis of *AciI* methylation patterns of integrated eGFP in C1 and D1 clone** *AciI* methylation pattern analysis in parental C1 clone (Fig. S10B and Fig. 7B) highlights methylation only in the d amplicon, whereas both c and e amplicons always resulted completely unmethylated (lanes 1, 4 and 7). In the d amplicon, the non fluorescent parental C1cells (lane 1) showed higher levels of methylation than fluorescent ones. SDF-modified and re-sorted fluorescent D1 clone (Fig. S10C and Fig. 7C) showed no methylation (c and e amplicons) in the fluorescent cells (lane 1), while non fluorescent D1 cells (lane 4) resulted methylated in all amplicons.
